# Modulating Integrin and Growth Factor Signaling With Peptides: Strategies to Synergistically Enhance Bone Tissue Regeneration

**DOI:** 10.1002/advs.202514782

**Published:** 2026-01-05

**Authors:** Lluís Oliver‐Cervelló, Elisabetta Ada Cavalcanti‐Adam, Carlos Mas‐Moruno

**Affiliations:** ^1^ Biomaterials Biomechanics and Tissue Engineering Group Department of Materials Science and Engineering and Institute for Research and Innovation in Health (IRIS) Universitat Politècnica de Catalunya Barcelona Spain; ^2^ Centre For the Cellular Microenvironment Advanced Research Centre University of Glasgow Glasgow UK; ^3^ Cellular Biomechanics University of Bayreuth Bayreuth Germany; ^4^ Barcelona Research Center in Multiscale Science and Engineering Universitat Politècnica de Catalunya Barcelona Spain; ^5^ Centro de Investigación Biomédica en Red – Bioingeniería Biomedicina y Nanomedicina (CIBER‐BBN) Madrid Spain

**Keywords:** BMP, integrin, growth factor, RGD, synergy

## Abstract

Providing biomaterials with cell‐instructive cues represents a major goal in tissue engineering to tailor cell‐material interactions and guide tissue regeneration. For bone tissue regeneration, this has been classically addressed by integrating integrin‐binding proteins and peptides on different substrates, aiming at mimicking the bone extracellular matrix. However, it has become evident that integrin signaling alone is not sufficient to fully recreate the cellular microenvironment required to effectively drive bone healing, and that growth factors (GFs), such as bone morphogenetic proteins (BMPs), are essential for bone development and growth. Indeed, recent research has demonstrated that integrins engage in synergistic signaling with GF receptors (GFRs). For instance, it is now well established that the osteogenic activity of BMPs can be regulated, and often enhanced, by integrins. These findings have thus encouraged the study of novel multifunctional systems that combine integrin‐binding ligands with osteoinductive cues to exploit integrin‐GF crosstalk and regenerate bone. This review aims to provide a comprehensive overview of the different mechanisms of signaling between integrins and GFRs, and the existing strategies to harness synergistic effects. A particular focus is put on the recent developments using the well‐known integrin‐binding RGD peptide and its co‐presentation with BMP‐derived peptides.

## Graphical abstract

It has been demonstrated that, in the bone extracellular matrix (ECM), integrins and growth factor receptors (GFRs) engage in synergistic signaling to guide bone healing and regeneration. This review provides a comprehensive overview of current strategies using ECM‐derived peptides to recreate the cellular microenvironment and harness synergistic effects on biomaterials.

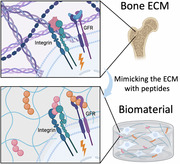

## 1 Introduction

Bone is a mineralized connective tissue with an outstanding capacity to self‐regenerate after injury. This unique property is mediated by a well‐orchestrated and dynamic process known as bone remodeling, and allows for the recovery of the original structure and function of the tissue. Nonetheless, physiological homeostasis and complete regeneration are hampered under certain musculoskeletal conditions. Moreover, in non‐union bone fractures or bone defects larger than a critical size, complete healing is not possible. In these cases, clinical intervention is required to ensure adequate regeneration of the tissue. Such a problem has been aggravated over the last few years by the increase in life expectancy and aging of the population, which is in turn associated with a number of age‐related diseases, such as osteoporosis, as well as the lower healing capacity of elderly patients. In fact, bone is the most transplanted tissue after blood, involving more than 2 million bone grafts annually [1]. Furthermore, osteoporosis affects more than 200 million people worldwide, and 1 in 3 women and 1 in 5 men will experience osteoporotic fractures in their lifetime [2]. For instance, a recent report estimated 4.3 million new fragility fractures in the European Union and an associated cost of €55.3 billion in 2019 [3, 4]. The current gold standard in bone tissue engineering is still the use of autografts, mainly due to their inherent osteoinduction capacity (i.e., they promote the differentiation of stromal cells into the osteoblastic lineage, thus triggering osteogenesis) [5], and the absence of adverse immunological reactions. However, there are some significant drawbacks associated with autologous bone grafting, including the limited quantity of graft available, patient morbidity, and the need for an additional surgery [6, 7]. A possible solution is the use of allografts or xenografts, but although they potentially overcome some of the limitations related to autografts, well‐documented risks of infection, disease, and graft rejection by the immune system exist for both cases [8].

Alternatively, synthetic human‐made biomaterials can be employed for this purpose, as such artificial materials are largely available, can be fabricated with defined geometries, and may be engineered to meet the desired properties for a specific application. Indeed, a gradual shift from natural grafts toward synthetic materials and biological factors has been recently noted, especially in the U.S. and European markets [9, 10]. However, a great deal of the currently available biomaterials, like synthetic polymers or metals and their alloys, lack osteoconductive (i.e., capacity to foster bone growth) and osteoinductive potential, and generally fail to stimulate the osteogenic differentiation of mesenchymal stem cells (MSCs) and support bone growth, required for complete healing and regeneration of the tissue. Moreover, the metallic implants used in load‐bearing applications may suffer from the stress‐shielding effect, leading to increased and undesired rates of bone resorption. A lower promotion of cell adhesion and growth on the biomaterial surface, as well as its limited vascularization, may eventually end up with the failure of the implant [11], which is catastrophic for the patient and poses a growing burden in the healthcare system. Most of these problems arise from the intrinsic properties of synthetic materials, in particular, their bio‐inertness, i.e., lack of bioactivity, which is essential to support the regenerative cascade of events necessary after implantation. Hence, the combination of biomaterials with biological cues that mimic the healing microenvironment and foster bone tissue regeneration has driven remarkable research efforts by the biomaterials community in recent years [12, 13, 14, 15, 16].

Thus, the aim of this review is to provide a comprehensive overview of the existing strategies to improve the bioactivity of biomaterials and their capacity to regenerate bone, based on the signaling mechanisms reported for both stem and bone cells. To this end, we will first introduce the characteristics of the healing microenvironment defined by bone extracellular matrix (ECM), with a particular focus on integrins and growth factor (GF) receptors (GFRs). A summary of major signaling mechanisms activated by these receptors will be presented, and special attention will be given to their crosstalk and synergistic interactions. Building on this, the combination of integrin‐binding ligands and GF‐derived osteogenic sequences to mimic and recreate the regenerative potential of bone ECM will be covered. Different types of functionalization strategies will be reviewed, with special emphasis on the use of the integrin‐binding RGD peptide together with bone morphogenetic proteins (BMPs) and derived peptides, with the final aim of demonstrating that synergistic integrin and GF signaling can effectively enhance cell adhesion and osteodifferentiation on biomaterial surfaces. We believe that such understanding will contribute to developing novel biomaterials with superior osteogenic properties to promote better rates of bone healing and regeneration.

## 2 Engineering Bone Extracellular Matrix

The ECM is a complex and structured fibrous network of non‐cellular components present in all connective tissues. It is locally produced by the cells, which secrete specific biomolecules that are assembled extracellularly. These components include proteins, proteoglycans, and glycosaminoglycans, which are arranged in a tissue‐specific three‐dimensional architecture that provides cells (and tissues) with defined topographical, structural, and elastic properties. The ECM also mediates the attachment, migration, proliferation, and differentiation of cells, and very efficiently sequesters GFs and other morphogens, acting as a reservoir of these types of molecules for the cells. The ECM has thus a crucial role in the physiology of both healthy and pathological tissues, controlling biological processes as diverse as inflammation, angiogenesis, and tissue morphogenesis. This plethora of functions is spatially and temporally controlled by a series of complex and dynamic interactions [17, 18, 19, 20]. Recapitulating these interactions is essential to recreate the healing microenvironment required to foster the regenerative process.

**FIGURE 1:**
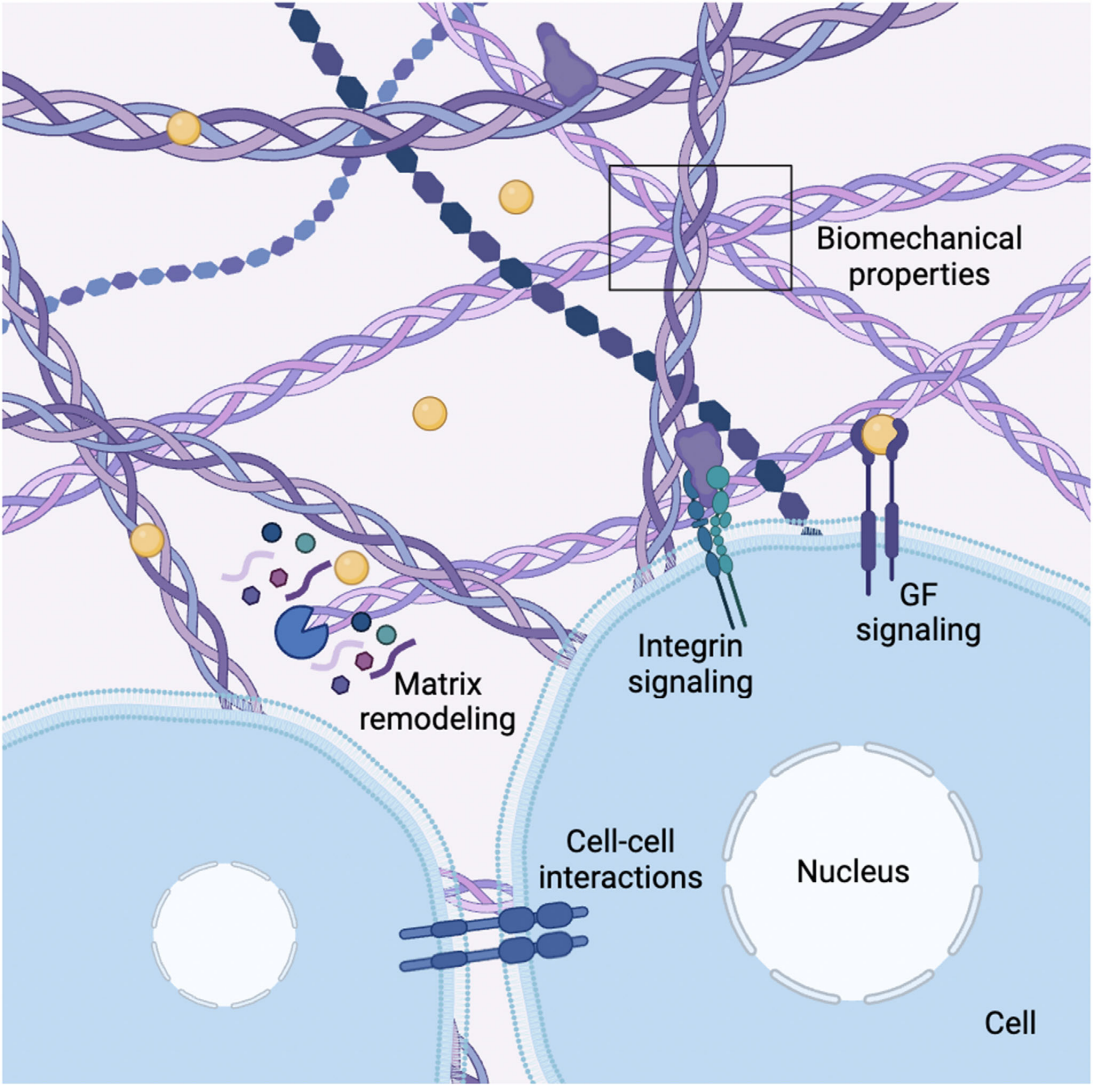
Schematic representation of the cellular microenvironment, highlighting major cell–ECM interactions and signaling cues involved in (bone) tissue regeneration. These include cell–cell interactions, integrin and GF activity, the stiffness and elasticity of the matrix, as well as protease‐sensitive sequences required for matrix remodeling. This review will focus on integrin and GF signaling.

In the case of bone, the complexity of the healing response has not been yet fully deciphered, but major aspects that have been identified and studied in bone cells include (i) cell–cell interactions, (ii) integrin signaling, (iii) GF signaling, (iv) the biomechanical properties of the matrix, and (v) degradation sites for matrix remodeling (Figure 1). Bone tissue healing microenvironment is regulated by a complex network of cues arising from ECM biomechanical properties, such as stiffness and elasticity, and its protease‐sensitive sites that facilitate matrix degradation and turnover. These cues are sensed and transduced by cell‐adhesion receptors, including integrins and syndecans, which form focal adhesions and participate in co‐signaling pathways. At the same time, soluble signaling molecules, such as GFs and cytokines, integrate with ECM‐derived signals to modulate intracellular pathways. This interplay drives nuclear responses and downstream gene expression, leading to further ECM remodeling through matrix synthesis and degradation. These processes, along with autocrine and paracrine signaling, regulate key aspects of cell fate such as proliferation, differentiation, migration, and apoptosis. Such dynamic feedback loops between ECM remodeling and cellular responses lead to a tuned microenvironment that allows effective tissue repair and regeneration.

This review will primarily focus on cell–ECM interactions mediated by integrins and GF receptors and the capacity of these two receptor types to engage in synergistic signaling.

### 2.1 Integrins

**FIGURE 2:**
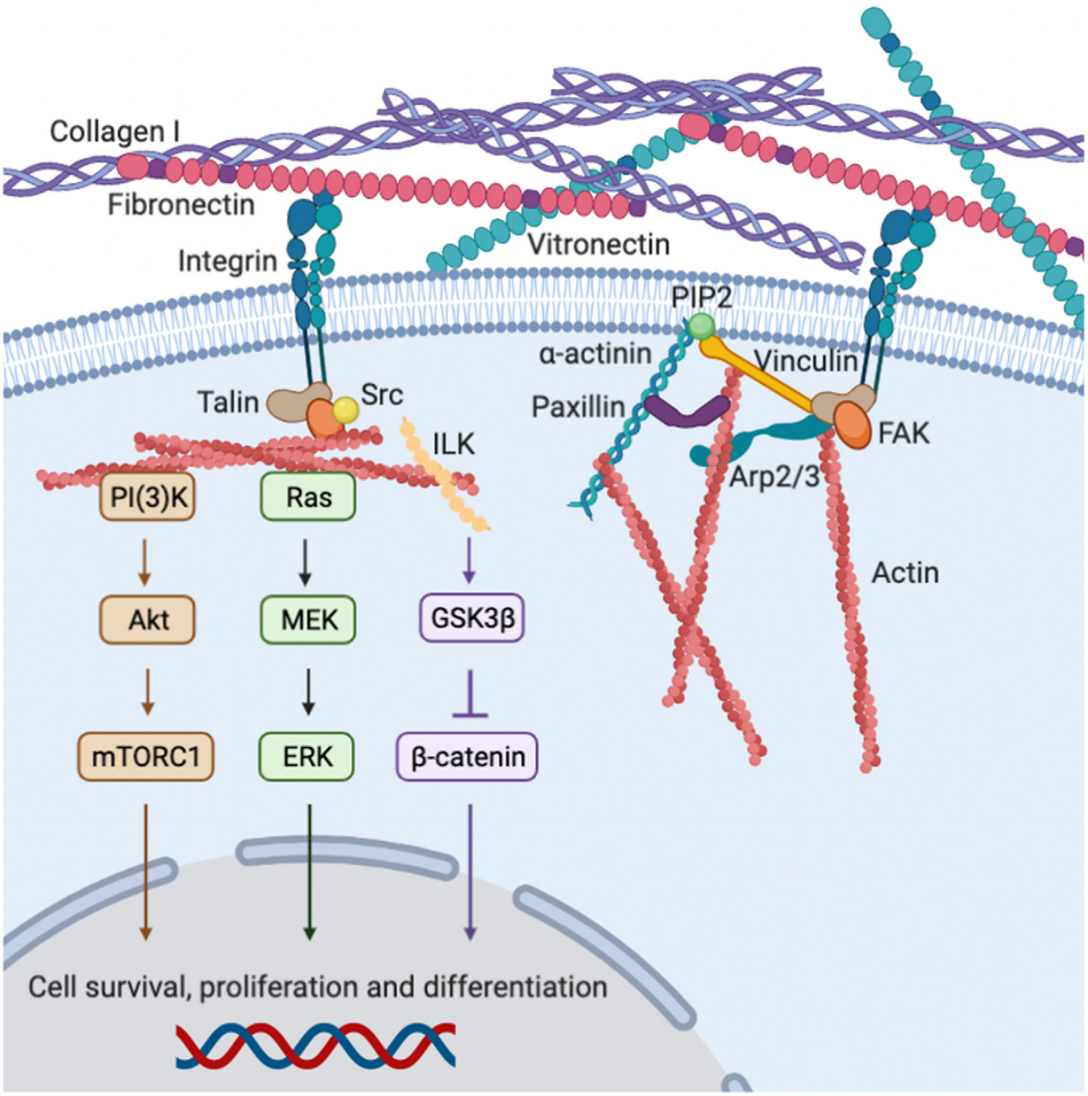
Representative signaling cascades triggered by integrin interactions with ECM ligands (left) and linkage between ECM proteins, integrins, and the actin cytoskeleton (right). The linkage between the ECM and the actin cytoskeleton is mediated by integrins and a series of cellular proteins, such as talin, vinculin, and α‐actinin. The activity of both vinculin and α‐actinin is modulated by phosphatidylinositol (4,5)‐bisphosphate (PIP2). Vinculin and FAK can also bind to the actin nucleator Arp2/3. Integrin‐mediated signaling activates different intracellular cascades, including PI(3)K‐Akt‐mTORC1 and Ras‐MEK‐ERK, promoting cell survival, proliferation, and differentiation. Finally, the integrin‐linked kinase (ILK) also modulates the GSK3β‐β‐catenin pathway, influencing gene expression and cellular fate decisions [24, 25].

The ECM is a dynamic and diverse scaffold composed of proteins such as collagen I, fibronectin (FN), and vitronectin (VN), which modulate cellular behavior through their interaction with integrins. Integrins, transmembrane receptors with specificity for distinct ECM ligands, not only anchor cells to their surrounding matrix but also serve as critical hubs for bidirectional signaling. Integrins are considered the most prominent family of cell adhesion receptors, playing crucial roles in a plethora of cellular functions in all higher organisms. They are composed of at least 24 subtypes, assembled by the non‐covalent association of 18 α and 8 β subunits. Upon ECM binding, integrins cluster and recruit intracellular focal adhesion proteins such as talin, vinculin, paxillin, and focal adhesion kinase (FAK), which link integrins to the actin cytoskeleton and enable mechanotransduction. These focal adhesion complexes act as signaling platforms, activating downstream pathways that regulate essential cellular processes, such as cell adhesion, morphology, proliferation, survival, migration, and differentiation. Such communication with the ECM is done in a bidirectional manner and requires their activation, as otherwise integrins are found in an inactive state (bent conformation) in which they do not bind ECM ligands and hence are unable to engage in signal transduction [21, 22]. For instance, integrins can respond to environmental cues. In this case, “outside‐in” signaling is produced when an ECM ligand binds to the extracellular domain of integrins, changing their conformation and directing intracellular responses such as cell polarity, cytoskeletal organization, and gene expression [23]. Conversely, during “inside‐out” signaling, cytoplasmic molecules can bind to the intracellular tail of integrin β subunits, activating the receptors and increasing their affinity for extracellular ligands (Figure 2), triggering cell adhesion and downstream signaling. For example, integrin‐mediated signaling can activate the PI(3)K‐Akt‐mTORC1 pathway, promoting cell survival and proliferation, while the Ras‐MEK‐ERK cascade governs proliferation and differentiation. Furthermore, integrin‐linked kinase (ILK) modulates the GSK3β‐β‐catenin pathway, influencing gene expression and cellular fate decisions. By integrating biophysical and biochemical signals from the ECM, focal adhesions enable cells to adapt dynamically to their microenvironment, contributing to tissue development, repair, and homeostasis.

Integrins are able to bind to specific sites of ECM proteins. Among all the available binding domains, the well‐known Arg‐Gly‐Asp (RGD) motif, present in FN and several other ECM proteins, represents the most prominent recognition motif involved in cell adhesion [26]. However, RGD is recognized with varying affinity by different integrins, and, to date, only less than half of the 24 known integrins have been reported to bind the RGD sequence [27, 28, 29]. Of relevance for bone tissue engineering, a particular subset of these integrins plays a key role in osteoblast adhesion and osteodifferentiation; namely, the α_5_β_1_ integrin, which mainly binds to FN, and the α_v_β_3_ integrin, the native VN receptor [30]. In this regard, the importance of these two integrins in the expression of osteogenic genes in MSCs has been widely documented [31, 32, 33, 34, 35]. In addition, the α_2_β_1_ integrin, through its interaction with collagen type I (the most abundant protein in bone ECM), has also been found to activate ROCK and MAPK ERK 1/2 signaling, both involved in the activation of osteogenic genes [36].

Based on the evidence mentioned above, integrin‐binding ligands have been widely used to engineer biomaterials with enhanced cell adhesive properties for bone regeneration. The canonical example, RGD, still represents the most frequently used peptide for this purpose [37]. Moreover, extensive work done by the group of Horst Kessler and others has shown that it is possible, by conformational restriction via cyclization, to increase the affinity of RGD for integrins α_v_β_3_ and α_5_β_1_, and even to discriminate between these two receptors by designing fully non‐peptidic RGD‐based peptidomimetics [30]. Another relevant sequence is GFOGER, which represents the minimal binding site in collagen for integrins α_2_β_1_ and α_1_β_1_ and has shown potential to promote osseointegration and bone regeneration in vivo [38, 39, 40]. However, the fact that this sequence needs to be presented in a triple helical conformation to exert its adhesive activity limits its use in shorter peptidic configurations, unlike RGD [41].

### 2.2 Growth Factors

Although cell‐adhesion ligands are important to mimic bone ECM and support cell adhesion and activation, soluble signaling molecules such as GFs are crucial and required to effectively drive tissue growth and regeneration. Indeed, GFs are biomolecules naturally secreted by cells that significantly contribute to regulating cell proliferation, migration, and differentiation [42]. The GF family is very large and is composed of a variety of proteins, including the epidermal growth factor (EGF), the fibroblast growth factor (FGF), the vascular endothelial growth factor (VEGF), and the transforming growth factor beta (TGF‐β), to name a few [43]. Among all of them, BMPs, which belong to the TGF‐β superfamily, have been described to induce the differentiation of MSCs into the osteoblastic lineage, which makes them essential in bone formation and remodeling, as well as in fracture repair [44, 45].

**FIGURE 3:**
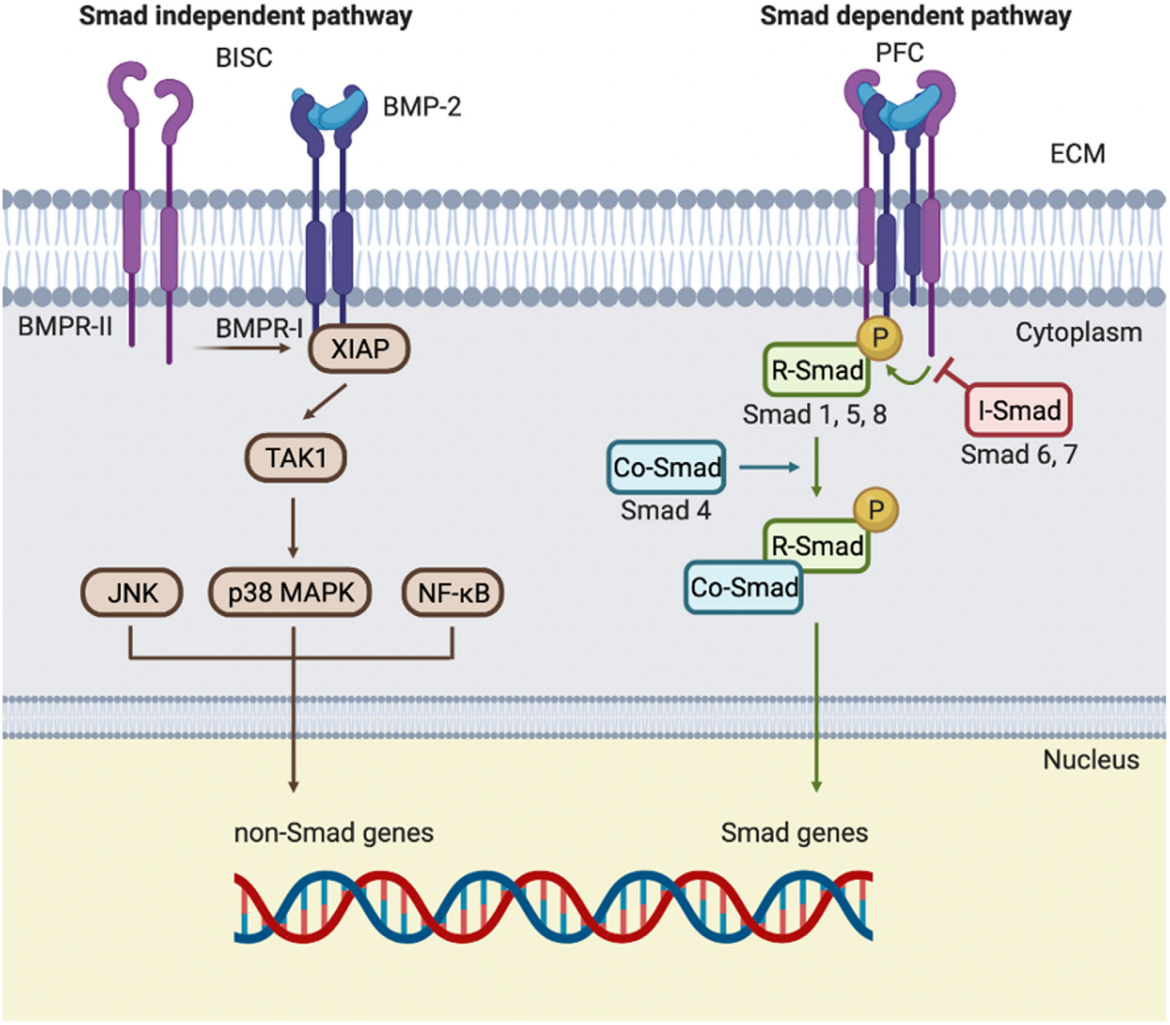
Schematic representation of BMP‐2 signaling cascades. BMPs interact with different serine/threonine kinase receptors, named BMPR‐I and BMPR‐II. The binding of BMP‐2 to a preformed complex (PFC) of these receptors leads to Smad‐dependent signaling (right). Alternatively, the formation of a BMP‐induced signaling complex (BISC) activates a Smad‐independent pathway (left). Both signaling mechanisms promote osteogenic gene expression required in bone formation.

BMPs interact with transmembrane serine/threonine kinase receptors, classified as bone morphogenetic protein receptors type I (BMPR‐I) and type II (BMPR‐II). Depending on their affinity for BMP receptors, as well as on the distribution of these receptors in the cell membrane, BMPs may bind either to a preformed complex (PFC), triggering a small mothers against decapentaplegic (Smad)‐dependent signaling, or to a BMP‐induced signaling complex (BISC), initiating, in this case, a Smad‐independent pathway. However, both cascades will end up stimulating transcription factors, which will enter the cell nucleus and activate osteogenic genes (Figure 3) [46, 47].

Currently, about 20 different types of BMPs have been identified [48]. Of these, BMP‐2, BMP‐4, BMP‐6, BMP‐7, and BMP‐9 have been demonstrated to be highly involved in bone formation [49, 50]. Nonetheless, only recombinant human BMP‐2 and BMP‐7 have been approved by the U.S. Food and Drug Administration (FDA) and the European Medicines Agency (EMA) for their clinical practice as osteoinductive graft substitutes [51]. For instance, BMP‐2 has been used in combination with a collagen type I sponge to treat anterior lumbar interbody fusions; whereas BMP‐7, also within a collagen sponge, has been administered for treating large bone defects, such as severe tibial fractures [52, 53, 54]. These reports thus highlight the potential of using BMPs to mimic the healing microenvironment of bone ECM.

However, BMPs (and GFs in general) need to be delivered at supraphysiological doses (typical clinical BMP‐doses range from 1–12 mg per implant) because of their short half‐life and clearance in vivo, and such high dosing regimen entails serious drawbacks. For example, BMP‐2 has been shown to induce ectopic bone formation, inflammation, osteolysis, and, in some contexts, tumor‐promoting effects. Indeed, the FDA issued a notification of the potential life‐threatening complications of BMP‐2. These limitations, together with the high cost and complex bio‐manufacturing of recombinant proteins, have raised controversy on the safe use of these types of molecules for bone tissue healing therapies, and underline the necessity of safer strategies that reduce BMP‐2 dosage or even replace their pharmaceutical use [55, 56].

### 2.3 Crosstalk Between Integrins and Growth Factor Receptors

As it has been previously shown, both integrin‐binding ligands and GFs play a relevant role in regulating the healing microenvironment of bone, particularly in terms of controlling cell adhesion to the ECM and osteogenic differentiation. Moreover, it has been demonstrated that integrins and GFRs can cooperate by different mechanisms of crosstalk, resulting in increased signaling, which may ultimately enhance the process of bone regeneration [57].

**FIGURE 4:**
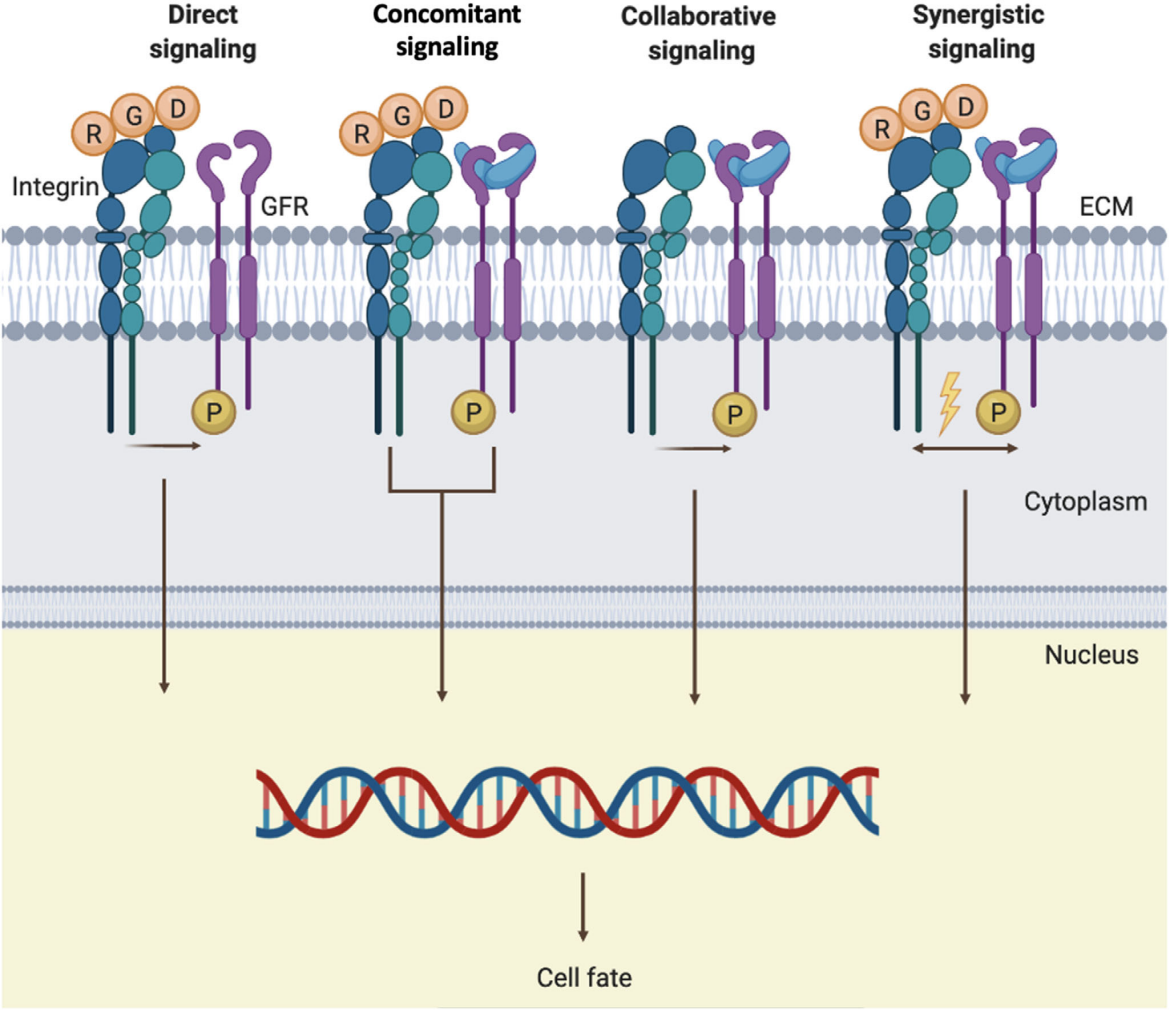
Interactions between integrins and GFRs, illustrating the most representative mechanisms identified. In the first case, integrins can activate GFRs even if GFs are not present *(direct signaling*); alternatively, integrin‐binding ligands and GFs can activate their receptors independently, but regulating the same downstream pathway (*concomitant signaling*); in another mechanism, integrins are required to enable GF‐dependent GFR signaling (*collaborative signaling*); and finally, the activation of integrins by ECM ligands in the vicinity of GF–GFR interactions triggers the formation of integrin–GFR complexes that result in receptor crosstalk and additive signaling (*synergistic signaling*) [64].

The precise mechanisms governing integrin‐GFRs interactions are still under investigation and not fully elucidated. For instance, it is not yet clear whether a spatial coexistence of integrins and GFRs is required to initiate their crosstalk, although it is thought that co‐localization facilitates it [58]. Nonetheless, the most representative described mechanisms are direct, concomitant, collaborative activation and synergistic signaling (Figure 4) [59]. In *direct signaling*, integrins may activate GFRs, even in the absence of GF binding. This is the case of the platelet‐derived growth factor (PDGF), which may be phosphorylated and thus activated by the α_5_β_1_ integrin without further stimulation of a GF [60]. In *concomitant signaling*, integrins and GFRs can activate the same signaling cascade independently. A good example is the activation of the Ras‐MAPK (mitogen‐activated protein kinase) pathway [61]. In the case of *collaborative signaling*, integrins are necessary to activate the GFR, even after GF binding. This means that once the GF has interacted with its receptor, integrins need to create a favorable environment to allow GFR downstream signaling. This type of signaling has been observed in cell adhesion survival, in which adherent cells depend on integrin‐mediated adhesion to be responsive to GFs [62]. Finally, in *synergistic signaling*, the binding of an integrin to an ECM protein, in a region close to GF‐GFR interactions, can initiate the formation of integrin‐GFRs complexes, resulting in receptor crosstalk and the consequent additive activation of signaling cascades, such as the interaction between integrin α_v_β_3_ and BMP‐2 to stimulate osteogenic genes [57, 63].

As a consequence, integrin–GFR interactions are of major importance to adequately mimic the interactions of cells with bone ECM. In this regard, extensive research trying to understand such interplay has been carried out, which has allowed the engineering of biomaterials with enhanced osteogenic potential [17, 57, 65, 66].

## 3 The Role of Fibronectin in Biomaterials for Bone Regeneration

FN is an ECM glycoprotein that assembles into a fibrillar matrix through a cell‐mediated process. As a key multifunctional constituent of the ECM, it encompasses different bioactive domains capable of establishing specific interactions with diverse elements, including other ECM proteins, glycosaminoglycans (GAGs), GFs, and cell adhesive receptors (e.g., integrins) [67]. In particular, FN‐integrin interactions occur through the RGD motif, located in the tenth type III module of FN (FN III10). Furthermore, the cell attachment site of FN is complemented with another peptide sequence (PHSRN), which is located at the FN III9 module, and synergistically enhances the affinity of RGD toward α_5_β_1_ integrin. This receptor is a major mediator of cell adhesion, and, as previously mentioned, it positively regulates osteoblastic differentiation [68, 69]. Based on these evidences, the specific engagement of α_5_β_1_ opened promising prospects to enhance biomaterials’ functionality in bone regeneration, as reported in several approaches (Table 1).

**TABLE 1:** Representative examples of the use of full‐length FN and recombinant FN fragments to engineer biomaterials for enhanced bone regeneration.

Bioactive motif(s)	Substrate	Biological outcome	Refs.
FN III7‐10	Titanium	↑ α_5_β_1_ interaction, osteogenic differentiation, osseointegration in vivo	[70, 71, 72]
	Stainless steel		[73]
FN III9*‐10	TCPS / fibrin matrices	↑ α_5_β_1_ interaction, osteogenic differentiation	[74]
FN III9‐10/12‐14 + GFs (e.g., BMP‐2)	Fibrin hydrogel	↑ α_5_β_1_‐GF synergy, sequester GFs, ↑ osteogenic genes, bone regeneration in vivo	[76]
FN III9*‐10 + BMP‐2	HA hydrogel	↑ Cell adhesion, bone regeneration, organization in vivo	[77]
FN III8‐10/12‐14	TiNbHf alloy	↑ Cell adhesion, osteogenic differentiation	[78]
FN III12*‐14 (+ BMP‐2)	Titanium disks	↑ Cell adhesion, osteogenic differentiation	[79]
FN + BMP‐2	PEA	↑ Osteogenic differentiation, bone regeneration in vivo	[80, 81]
	Glass substrate	↓ Myotubes, ↑ Smad signaling	[83]
	PLL‐HA film	↑ Smad signaling	[84]
FN + rLTBP1	PEA	↑ Osteogenic differentiation, bone regeneration in vivo	[82]
FN + OGP	Titanium + mineral phase	↑ Cell adhesion, proliferation, and osteogenic differentiation	[85]

A representative example of this strategy is illustrated by the pioneering work of Garcia's group, which focused on the design of recombinant protein fragments recapitulating the cell attachment site of FN to develop biomimetic coatings. In particular, titanium implants were coated with an antifouling oligo(ethylene glycol)‐substituted polymer, in which a FN III7‐10 fragment was tethered to address α_5_β_1_ integrin interactions. Such a strategy showed a significant improvement in the osteodifferentiation of bone marrow stromal cells, in comparison with bare titanium and RGD‐functionalized controls, which mainly bound to α_v_β_3_ integrin. Furthermore, in vivo results highlighted an enhancement of bone healing and osseointegration on titanium implants modified with FN III7‐10 [70]. Of note, similar findings, both in vitro and in vivo, were observed when titanium surfaces were directly coated with the same recombinant fragment by simple physisorption, without using the antifouling polymer [71]. Founded on these results, the same strategy was followed to functionalize titanium surfaces, but using dimers, trimers, or pentamers of FN III7‐10, instead of single monomers. The rationale was thus to evaluate the biological effect of ligand clustering at the nanoscale level. Interestingly, a significant increase in integrin α_5_β_1_ affinity toward the trimers and pentamers was noticed, which was associated with higher alkaline phosphatase (ALP) activity and mineralization of MSCs. The promotion of bone formation and osseointegration on the trimer‐ and pentamer‐coated implants in vivo further supported the importance of ligand nanoclustering to elicit bone regeneration [72]. In addition, FN III7‐10 coating of stainless steel by passive adsorption also improved the number of adherent MSCs, mediated by α_5_β_1_ integrins, their projected area, as well as their osteogenic differentiation. Of note, when coating stainless steel screws and implanting them into osteoporotic rats, the bone screw‐fixation and the bone‐implant ingrowth both increased about 30% after 3 months of implantation, compared to uncoated screws [73]. These studies highlight the potential of mimicking integrin signaling on biomaterial surfaces to enhance their osteogenic potential, even under challenging scenarios, such as those present in osteoporotic bone.

Following a similar rationale, Martino et al. took the FN III9‐10 fragment and compared it to FN III9*‐10 (in which Leu^1408^ was mutated to Pro) and FN III10 alone (i.e., RGD). Studies in 2D models showed that the mutated fragment, which enhances the conformational stability of the original fragment, displayed a higher specificity toward α_5_β_1_, resulting in an increased induction of osteoblastic gene expression and ALP activity. Similar results were observed in 3D environments using fibrin matrices [74]. Subsequently, in another work, they integrated in a single polypeptide chain FN III9‐10 with FN III12‐14 (previously shown to be a promiscuous GF‐binding domain)[75] within a fibrin hydrogel. The results showed the capacity of the matrix to sequester diverse GFs, including BMP‐2 and PDGF, inducing the expression of osteogenic markers, as well as a synergistic signaling between integrins and GF receptors in vivo [76]. This work was relevant in demonstrating that the crosstalk observed between these receptors at the cell level could be used to engineer material‐based systems with enhanced regenerative profiles. Moreover, these systems showed the potential to exploit synergistic signaling with very low doses of GFs, paving the way to develop safer and more efficient GF‐based strategies in the clinic. For instance, in a subsequent work, the same authors showed that the covalent grafting of the structurally stabilized FN III9*‐10 fragment into a hyaluronic acid (HA) hydrogel improved the osteogenic potential of BMP‐2. With more detail, the functionalized HA hydrogel improved MSC attachment and spreading in vitro, compared to the low‐adhesive non‐functionalized HA. Moreover, loading this hydrogel with BMP‐2 resulted in the formation of two times more bone in an ectopic bone formation model in vivo, compared to the delivery of the same GF within the unmodified HA hydrogel, highlighting once again the synergistic potential of BMP‐2 and integrin‐binding ligands [77].

Alternatively, Guillem‐Martí et al. combined the FN III8‐10 and FN III12‐14 fragments in different proportions to covalently functionalize TiNbHf alloys. Of note, MSC spreading and proliferation were enhanced on samples coated with a mixture of the two fragments containing 50% (or less) of FN III8‐10, compared to samples coated with RGD or the individual fragments. In contrast, the presentation of the FN III12‐14 fragment alone or at high proportions (higher than 50%) stimulated the highest MSC adhesion and ALP activity, demonstrating the importance of controlling the rate of different FN fragments to target a particular function [78]. Later on, building on the described capacity of the FN III12‐14 fragment to attract GFs, the same research group designed a new fragment mutating the P^233^G^234^V^235^ region into R^233^G^234^D^235^, thus incorporating to FN III12‐14 cell adhesion capacity as well (i.e., the RGD sequence). Indeed, an improvement of the projected area of MSCs was noticed on the samples coated with the mutated fragment, compared to controls, while maintaining the GF‐binding capacity of the fragment and its osteodifferentiation potential, as demonstrated by an enhanced ALP activity, mineralization, and expression of osteogenic genes [79].

**FIGURE 5:**
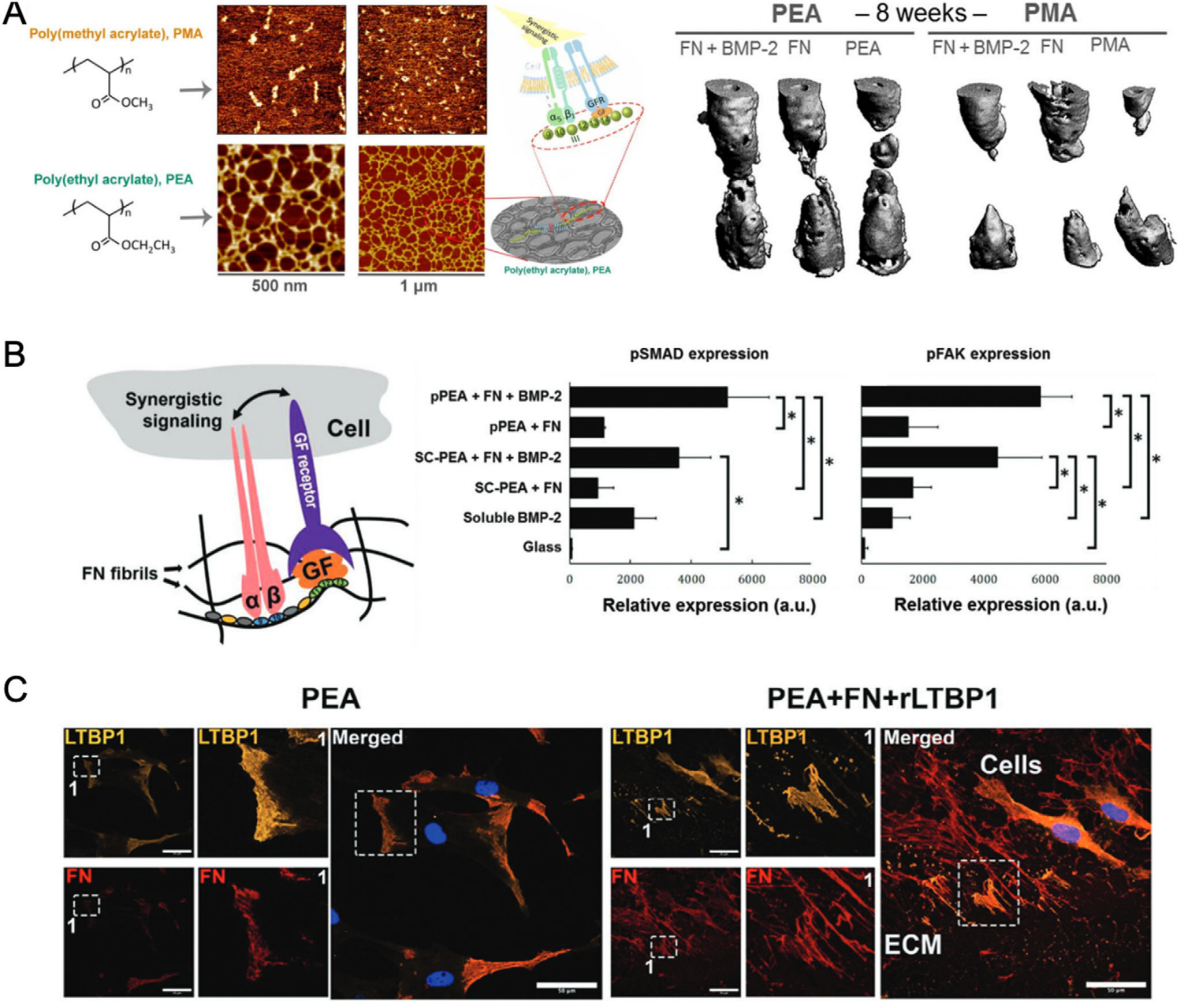
Summary of representative works using PEA to control FN fibrillar organization and the efficient presentation of GFs. (A) Comparison of the FN conformation on PMA (globular conformation) vs. PEA (fibrillar conformation) surfaces and bone regeneration potential of each strategy to heal critical sized bone defects in vivo. Reproduced from [80] (Creative Commons CC BY license) (B) Synergistic signaling between integrin and GF receptors occurs when the respective binding domains of FN are in close proximity and available for the cells. The crosstalk between adhesion and GF pathways is demonstrated by the expression of pFAK and pSMAD, respectively. Reproduced from [81] (Creative Commons CC BY license) (C) FN‐coated PEA surfaces were employed to immobilize rLTBP1. This strategy promotes the binding of LAP and the subsequent, integrin‐mediated, release and activation of TGF‐β1. Reproduced from [82] (Creative Commons CC BY license).

The capacity of FN to interact and sequester diverse GFs, such as BMP‐2, has been paramount to developing novel material platforms that synergistically promote cell adhesion and osteodifferentiation. In this regard, Salmerón‐Sánchez and co‐workers engineered a system based on the capacity of poly(ethyl acrylate) (PEA) to spontaneously drive the fibrillar organization of FN (Figure 5A). Noteworthy, they found that such conformation efficiently exposes both the integrin‐binding (FN III9‐10) and GF‐binding (FN III12‐14) sites of the protein, thus triggering synergistic integrin α_5_β_1_‐BMP‐2 signaling, which was translated into enhanced MSC osteodifferentiation and complete repair of non‐healing bone defects, notably using reduced BMP‐2 doses (i.e., ∼15 ng, which is a ∼300‐fold lower dose than the clinical standard using a collagen sponge) [80]. Of note, bone regeneration was not observed on control poly(methyl acrylate) (PMA) substrates, on which FN adopts a globular conformation where the integrin and GF‐binding motifs are hidden and not well‐exposed to interact with cell receptors. Following the same rationale, the same research group developed a plasma‐polymerization method for coating 3D scaffolds with thin layers of PEA, which allowed the delivery of ultralow doses of BMP‐2 in synergy with the integrin‐binding regions of FN. As previously reported, such synergistic signaling between integrin and GF receptors occurs when the binding domains (III9‐10 and III12‐14, respectively) of FN are in close vicinity (Figure 5B). Enhanced cell signaling in vitro was characterized by the colocalization of BMP receptor type 1A (BMPR1A) with focal adhesions and the crosstalk between adhesion and GF pathways, as illustrated by the increased expression of both phosphorylated SMADs (pSMAD) and phosphorylated focal adhesion kinase (pFAK). In vivo, this strategy showed excellent healing in a critical‐sized bone defect in mice and in a nonhealing humerus fracture in a Münsterländer dog, providing a strong ground for the clinical translation of using ultralow doses of GFs in synergy with integrin‐binding ligands [81]. Very recently, they also reported an unconventional approach to capture inactive GFs and their subsequent integrin‐mediated release and activation. This was achieved using FN‐coated PEA surfaces, which were engineered to immobilize a recombinant fragment of latent transforming growth factor beta‐binding protein‐1 (rLTBP1) (Figure 5C). This, in turn, promotes the binding of latency‐associated peptide (LAP), where an inactive version of TGF‐β1 is trapped. Successively, the mechanical pulling of LAP by integrin β_1_ and FN‐bound rLTBP1 triggers the release of TGF‐β1, thereby activating osteogenic TGF‐β1 signaling and bone regeneration in vivo [82]. The main novelty of this study lies in the fact that it presents a promising alternative to the administration of active GFs, as the capture of inactive GFs and their site‐specific activation would overcome a major challenge in the clinics.

In another approach, Hauff et al. exploited the co‐presentation of BMP‐2 non‐covalently bound onto microcontact printed FN stripes. They observed that the grafted BMP‐2 significantly inhibited myotube formation of C2C12 myoblasts and activated the Smad signaling pathway, as shown by Smad 1/5 phosphorylation and nuclear translocation, in a similar manner to soluble BMP‐2. Furthermore, they studied how the immobilization of BMP‐2 to FN bands fabricated by soft lithography influenced cell migration, concluding that the presence of BMP‐2 increased cells’ net displacement, and showcasing the versatility of this strategy to immobilize and retain the GF's bioactivity [83]. Following a similar rationale, Fitzpatrick et al. fabricated cell‐sized micropatterns of FN/BMP‐2 on soft polymeric substrates to study myoblast actin organization around the nucleus and the consequent Smad signaling. Indeed, the authors found that such cytoskeleton organization triggered the phosphorylation and nuclear translocation of Smad 1/5/8 in myoblasts through the BMP‐2‐dependent ROCK‐LIMK2 pathway [84]. Apart from BMP‐2, the osteogenic growth peptide (OGP—ALKRQGRTLYGFGG), which is known to stimulate bone healing, has been used together with FN too. In particular, titanium surfaces were coated with a mineral phase, which was later loaded with FN and OGP. The dual loading of the molecules synergistically fostered the adhesion, proliferation, and osteogenic differentiation of MSCs compared to FN or OGP loadings alone [85].

## 4 Co‐Presentation of RGD Peptides With BMPs for Bone Regeneration

The previous section showcased that the combination of integrin‐binding proteins of the ECM with GFs not only improves cell adhesion and osteodifferentiation but also may trigger the crosstalk between integrins and GF receptors, which can be translated into synergistic signaling, boosting osteogenesis. However, the cell attachment site of FN and many other ECM proteins can be recapitulated by the smaller, synthetically accessible RGD sequence. The advantages of using synthetic peptides, compared to full‐length proteins, are diverse and include higher stability, increased coating densities, lower production costs, and ease of handling [41]; based on this, an alternative body of research has focused on the co‐presentation of the RGD motif with BMPs (Table 2).

**TABLE 2:** Representative examples of the combination of RGD peptides with BMPs and major biological outcomes reported.

Bioactive motifs	Substrate	Biological outcome	Refs.
RGD + BMP‐2	PEG hydrogel	↑ Osteoconduction and bone formation in vivo	[86]
	NiPAM/NASI polymer	↑ C2C12 adhesion, ALP activity	[87]
	PLGA microspheres	↑ Osteogenic differentiation	[88]
	PCL mesh/alginate gel	↑ Bone volume, connectivity, and mechanical properties in vivo	[89]
	Alginate hydrogel	↑ ALP activity	[90]
	Oxidized alginate hydrogel	↑ Osteogenic differentiation, density of bone in vivo	[91]
	Chitosan/alginate on the titanium surface	↑ Cell adhesion, proliferation, ALP activity, and bone formation in vivo	[92]
	PEI/hydroxyapatite on titanium disks	↑ Cell adhesion, osteogenic differentiation	[93]
	Collagen sponge	↑ Ectopic bone formation, calcification in vivo	[94]
cRGD + BMP‐2	Gold nanopatterns	↑ α_5_β_1_ interaction, recruitment of β_3_ in focal adhesions	[96]
cRGD + BMP‐6	Gold nanopatterns	↑ Smad signaling, ↓ myotubes	[97]
CGGNGEPRGDTYRAY + BMP‐2	PCL films	↑ Actin organization, FAK, and Smad signaling	[98]
CGGNGEPRGDTYRAY + BMP‐2 and BMP‐9	PCL film	↑ Smad signaling, ALP activity	[99]
CGGPHSRNGGGGGGRGDG + BMP‐9	PCL film	↑ Cell adhesion, FAK, MAPK, Smad signaling, Runx2 expression	[100]
RGD + BMP‐7	Zirconia scaffold	↑ Osteogenic differentiation and bone formation	[101]

The literature is rich in examples of works integrating RGD with BMPs. For instance, PEG hydrogels containing RGD and matrix metalloproteinase (MMP)‐sensitive linkers were used to deliver BMP‐2 to critical‐sized defects in rats. Of note, it was observed that cells were able to colonize the scaffold and to produce new bone within 5 weeks of implantation [86]. Moreover, the process of bone regeneration was dependent on the MMP‐mediated proteolytic activity of the cells, representing an excellent example of a dynamic, stimuli‐responsive osteogenic material. In another example, RGD was immobilized on thermoreversible *N*‐isopropylacrylamide (NiPAM) polymers modified with *N*‐acryloxysuccinimide (NASI). RGD‐functionalized NiPAM/NASI improved the adhesion of C2C12 cells compared to non‐functionalized polymers. Subsequently, treatment of these cells with BMP‐2 induced a dose‐dependent expression of ALP [87]. However, in this study, a synergistic activity between RGD and BMP‐2 was not observed, and the authors concluded that the NiPAM/NASI surface was inherently osteoinductive, regardless of RGD grafting or BMP‐2 exposure. This lack of synergy might result from inadequate control over ligand presentation, as co‐presentation of RGD and BMPs has been shown to require precise spatial and density tuning to fully exploit integrin‐BMPR signaling crosstalk.

Alternatively, Park et al. engineered RGD‐modified microspheres of poly(lactic‐co‐glycolic acid) (PLGA) containing BMP‐2 and dexamethasone on their surface, which were used as a delivery vehicle on MSCs. By means of RT‐PCR and western blot, they detected the activation of specific genes and proteins related to bone regeneration when RGD and BMP‐2 were combined. Mineralization and in vivo assays further confirmed the capacity of such micro‐structured multicomponent scaffolds to induce osteodifferentiation [88]. Likewise, nanofiber meshes of poly‐(ε‐caprolactone) (PCL) were combined with RGD‐functionalized alginate gels, and loaded with different doses of BMP‐2 to be used as delivery systems. Implantation of such scaffolds in rat bone defects showed the dependence of bone healing as a function of BMP‐2 dose. In particular, bone volume, connectivity, and mechanical properties were improved with the hybrid system, compared to the clinical standard collagen sponge using the same dose of BMP‐2 [89]. This study highlights the importance of BMP‐2 concentration and spatial control of its delivery, as localized or immobilized BMP‐2 has been shown to activate Smad signaling more efficiently than soluble BMP‐2.

The importance of BMP presentation mode was also highlighted in a study, where RGD‐grafted alginate hydrogels delivering BMP‐2 from the bottom of cells (“bottom–up” release) were more efficient at inducing ALP activity in MSCs compared to top–down systems [90]. Such findings underscore the importance of spatial control over BMP release in optimizing osteogenic responses. Priddy et al. also developed BMP‐2 carriers using oxidized RGD‐functionalized alginate hydrogels. They demonstrated that the hydrogel was still bioactive after 26 days in culture with pre‐osteoblastic cells. Furthermore, such a biomaterial was able to foster the formation of more robust and dense bone in vivo than the controls [91]. Nonetheless, in these studies, RGD is generally used mainly as a cell adhesive factor, and the possible synergistic effects between RGD and BMP‐2 were not explored.

On the other hand, a synergistic response was described by Wang et al. following a different approach. In their study, the authors tried to mimic bone ECM by depositing layer‐by‐layer self‐assembled chitosan with oxidized RGD‐modified alginate on titanium substrates, which were subsequently loaded with chitosan‐coated bovine serum albumin (BSA) nanoparticles containing BMP‐2. The results indicated that the RGD grafted to alginate enhanced MSC adhesion and proliferation. Moreover, these nanostructures were able to control the release of BMP‐2 and, in the presence of RGD, synergistically promoted MSCs’ ALP activity as well as bone formation in vivo [92]. Titanium substrates were also coated with RGD‐functionalized poly(ethyleneimine) (PEI) in combination with hydroxyapatite nanoparticles and BMP‐2, in a process assisted by dopamine polymerization. As in the previous study, the introduction of RGD on the polymer improved cell adhesion, while the hydroxyapatite nanoparticles induced the switch of MSCs into osteoblast cells. Interestingly, the addition of BMP‐2 further enhanced such differentiation [93]. This multifunctional coating, combining RGD, hydroxyapatite, and BMP‐2, thus provided cell adhesive, osteoconductive, and osteoinductive properties to titanium. In addition, collagen scaffolds have also been used for a similar purpose. However, collagen functionalization with RGD usually requires chemical manipulations. Visser et al. engineered a “collagen‐targeted RGD biomimetic peptide” (WREPSFMALSGRGDS) by incorporating a sequence derived from the von Willebrand factor with collagen‐binding activity to the RGD sequence. Such a polypeptide was able to directly bind to absorbable collagen type I sponges, and the addition of low doses of BMP‐2 into the system elicited ectopic bone formation and enhanced the calcification levels of the scaffolds in vivo [94]. Interestingly, such effects were not observed when non‐functionalized collagen sponges were used with the same concentration of BMP‐2, suggesting that the synergistic effect of RGD and BMP‐2 is helpful to reduce the doses of GF currently used in surgical procedures.

**FIGURE 6:**
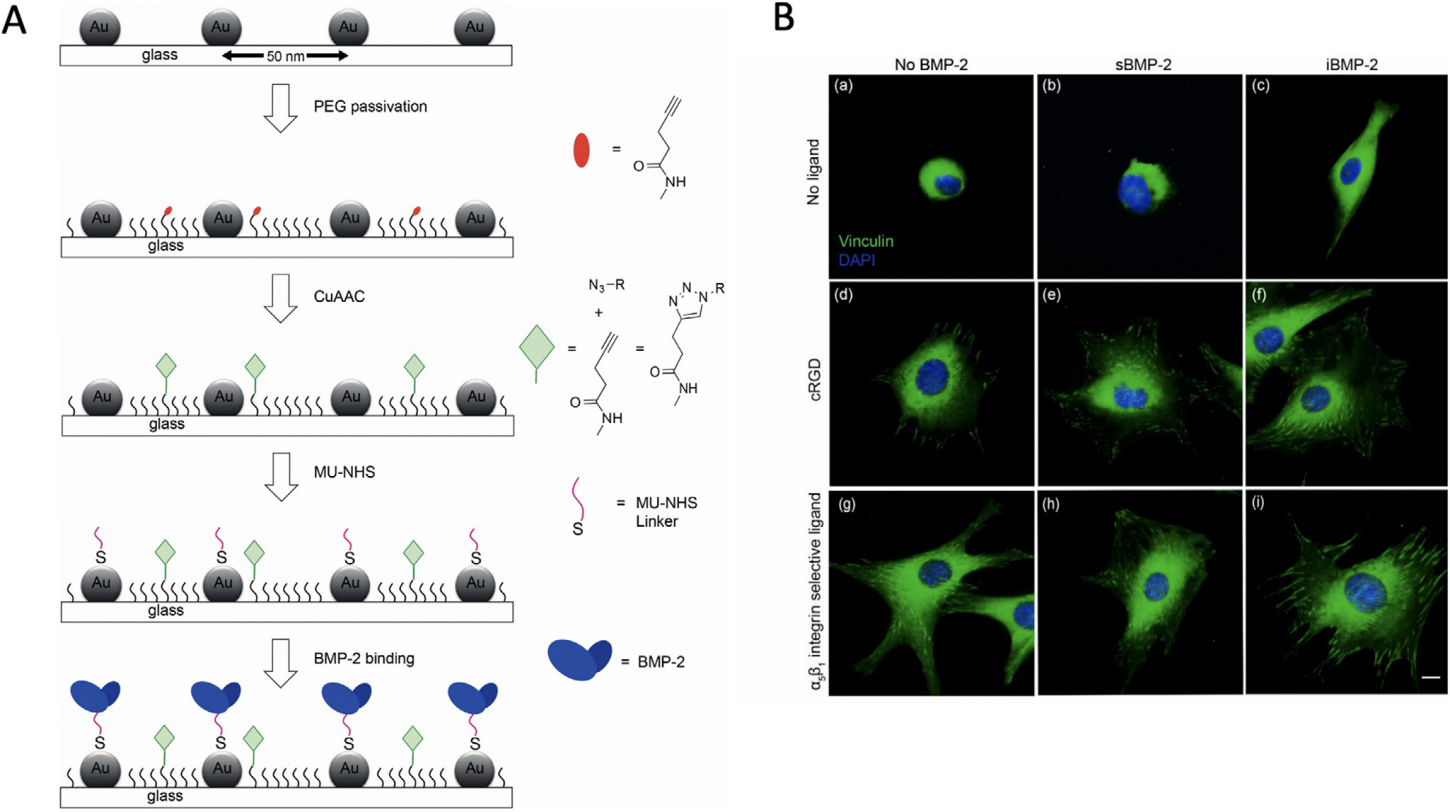
Co‐immobilization of integrin‐binding ligands and BMP‐2 using block copolymer micellar nanolithography (BCMN). Reproduced from [96] (Creative Commons CC BY license). (A) Azide‐containing integrin ligands (green) are bound to PEG‐alkyne end groups (red) via click chemistry, whereas BMP‐2 is attached onto gold nanodots functionalized with a heterobifunctional linker (11‐mercaptoundecanoyl‐N‐hydroxysuccinimide ester, MU‐NHS). (B) Formation of focal adhesions in cells adhering to nanopatterned surfaces functionalized with either cyclic RGD (cRGD) or an α5β1‐selective ligand in the presence or absence of BMP‐2. The study compared the use of soluble (sBMP‐2) or immobilized (iBMP‐2) GF. Reproduced from [96] (Creative Commons CC BY license).

In general, the majority of studies described so far rely on the use of polymers functionalized with RGD, which serve as substrates for BMP‐2 encapsulation. These systems, therefore, are merely used as GF delivery systems, and, notwithstanding the positive outcomes obtained in vivo, generally fail to control the grafting density and geometrical distribution of both RGD and BMP‐2, thus not allowing for the study of synergistic signaling in detail. To address this, the group of Cavalcanti‐Adam developed nanopatterned surfaces to covalently immobilize integrin‐binding ligands and BMP‐2 by using block copolymer micellar nanolithography (BCMN) [95, 96]. With more detail, they were able to selectively attach BMP‐2 onto gold nanodots via a heterobifunctional linker, whereas either cyclic RGD or an α_5_β_1_‐seletive peptidomimetic was grafted to the PEG passivating layer deposited on the rest of the surface by means of click chemistry (Figure 6A) [96]. The co‐presentation of BMP‐2 and cyclic RGD in such a spatially controlled manner resulted in the enlargement of focal adhesions, a hallmark of robust integrin clustering and activation (Figure 6B). Enlarged focal adhesions serve as critical sites for integrin‐mediated mechanotransduction, where signaling molecules such as talin, vinculin, paxillin, and FAK are recruited, enabling stronger anchorage to the ECM and enhanced cytoskeletal organization. This effect was particularly evident with the α5β1‐selective ligand, which reinforced adhesion selectivity by preferentially engaging integrin α5β1—a subtype crucial for osteodifferentiation. The enhanced integrin specificity not only promoted focal adhesion stability but also amplified downstream signaling, such as Smad‐dependent phosphorylation (Smad1/5/8) and Smad‐independent pathways (e.g., MAPK/ERK), both of which are vital for osteogenic gene expression [96]. The same research group also applied the previous technique to examine the influence of cyclic RGD with BMP‐6 on cell behavior. The co‐immobilization of both molecules in a spatially controlled manner promoted focal adhesion formation and Smad signaling (i.e., Smad 1/5/8 phosphorylation), and suppressed myotube formation of C2C12 myoblasts [97]. Of interest, it was again observed that Smad signaling was more efficiently induced by immobilized BMP‐6, compared to its soluble administration, which further emphasizes the importance of tuning ligand density and its presentation to achieve optimal cell signaling.

Although RGD was initially identified as the minimal cell‐binding motif in FN, this integrin‐binding sequence is also found in many other proteins of the ECM. Thus, other works have explored different RGD‐containing sequences in combination with BMP‐2. For example, the group of Faucheux studied the influence on cell behavior of PCL films functionalized with a bone sialoprotein (BSP) peptide (CGGNGEPRGDTYRAY), which contains the RGD sequence and is recognized by α_v_ integrin subunits and α_v_β_3_. The authors found that the presence of this peptide on PCL improved the organization of the actin cytoskeleton of preosteoblastic cells and activated intracellular signaling, as evidenced by FAK phosphorylation, compared to non‐functionalized PCL or PCL controls modified with a peptide containing RGE. Moreover, the cells responded to the addition of BMP‐2 by activating the Smad pathway (i.e., increased phosphorylation of Smad 1/5/8) [98]. Later on, they employed the same PCL substrates but loaded them with either BMP‐2, BMP‐9, or both proteins. They looked at the influence of the GFs in signal transduction, and although both GFs were able to translocate phosphorylated Smad to the nucleus, BMP‐2 induced earlier Smad phosphorylation than BMP‐9. After 2h of incubation, cells treated with BMP‐9 expressed lower β‐catenin, indicating that β‐catenin is a key factor in BMP‐2 signaling. Interestingly, mixing BMP‐2 and BMP‐9 maintained the β‐catenin levels constant, although it decreased the phosphorylated Smad signaling after 4h, showing a similar behavior to the use of BMP‐2 alone. Cells treated with both BMPs also expressed the highest osteocalcin (OCN) and ALP activity levels [99]. In another work, the same research group compared the effects of functionalizing PCL films with either the BSP‐derived peptide (which recognizes α_v_β_3_) or with a FN‐derived peptide containing the RGD and PHSRN sequences (CGGPHSRNGGGGGGRGDG) (which recognizes α_5_β_1_). In this case, they incorporated soluble BMP‐9 (and two peptidic analogues) and evaluated the capacity of the resulting systems to enhance cell adhesion and modulate mitogen‐activated protein kinase (MAPK) signaling. Interestingly, PCL films modified with the FN peptide were more successful in promoting cell cytoskeleton organization, FAK activation, and MAPK signaling than the surfaces coated with the BSP peptide. Moreover, the combination of the materials coated with the FN peptide together with soluble BMP‐9 (and related analogues) better fostered Smad 1/5/8 phosphorylation and the expression of Runx2, which is essential for cells to differentiate toward osteoblastic lineage [100]. The notable differences between these two peptides can be attributed to their distinct affinity for integrins α_v_β_3_ or α_5_β_1_, and would point out toward a more prominent role of α_5_β_1_ to exert integrin‐GF signaling, as also evidenced in the work done in the field using FN in combination with BMPs and other GFs (see Section 3 of this review); nonetheless, further research is warranted to clearly elucidate these differences in integrin activation and crosstalk.

## 5 Co‐Presentation of RGD With Cell Instructive Peptides for Bone Regeneration

As previously indicated, the potential use of ECM proteins and GFs for bone regeneration is limited by the inherent drawbacks associated with the use of these types of molecules. Proteins, in general, are very sensitive to changes in pH, temperature, and solvents, and are susceptible to enzymatic degradation. Furthermore, their production is time‐consuming, and even if purification methods are well established, the presence of contaminants and bacterial endotoxins may cause infection and induce adverse immunological reactions [41, 65]. In the particular case of GFs, an additional limitation is that their rapid clearance in vivo requires the use of elevated doses, which is associated with serious side effects [55, 56]. To avoid these shortcomings, in Section 4, we presented alternative strategies to the use of ECM proteins, mainly centered on the use of synthetic RGD. However, this strategy can also be applied to GFs. Following the same rationale applied to ECM proteins, it could be possible to reproduce the osteogenic potential of BMPs by recapitulating the epitopes involved in the interaction with BMP receptors with shorter peptides. As synthetic peptides are cheaper to produce and can be tailored to functionalize a wide variety of materials, often displaying similar biological potency to native proteins, this strategy has tremendous opportunities in the field [51, 102, 103].

Thus, in this section, we will first shortly describe the use of RGD combined with other sequences from the ECM that may synergistically enhance the cell adhesive properties of RGD and stimulate osteo‐differentiation (Table 3). Next, we will more specifically focus on the possibility of using RGD and BMP‐derived peptides (Table 4), aiming to achieve integrin‐GF signaling and enhanced osteogenic properties without the use of GFs, thus potentially overcoming the adverse effects associated with GF‐based therapies.

**TABLE 3:** Combination of the RGD motif with other cell adhesive peptides and major biological outcomes reported. The ECM protein (or tissue) from which these additional sequences are derived is indicated in brackets. The table also highlights when the geometrical disposition of the peptides was controlled.

Bioactive motifs	Substrate	Biological outcome	Geometry controlled	Refs.
RGD + FHRRIKA (BSP)	Quartz substrate	↑ Cell adhesion, mineralization	—	[104]
	Titanium disks	↑ Cell adhesion	—	[106]
			Yes	[108]
	Hydroxyapatite disks	↑ Cell adhesion ( = vs. RGD)	—	[107]
RGD + KRSR (BSP, FN, VN, …)	Titanium disks	↑ Cell adhesion	—	[106]
	Nanofibrillar hydrogel copolymer	↑ Cell adhesion, metabolic activity	—	[110]
	Hydroxyapatite disks	↑ Cell adhesion ( = vs. RGD)	—	[107]
	Silk nanofibers	↑ Cell adhesion, proliferation (no synergy)	—	[111]
	Titanium disks + PLL‐g‐PEG	↓ osteogenic differentiation	—	[112]
	Titanium implants + PLL‐g‐PEG	= BIC, bone fill, interfacial shear strength in vivo	—	[113]
	Titanium disks	↑ Cell adhesion, mineralization	Yes	[114]
RGD + PHSRN (FN)	TCPS	↑ Cell adhesion, MAPK signaling	Yes	[116]
	PEG hydrogel	↑ Cell adhesion, proliferation, and ALP activity	Yes	[117]
	Alginate hydrogel	↑ Cell adhesion, differentiation	—	[118]
	Titanium disks	↑ Cell adhesion, proliferation, and ALP activity	—	[119]
		↑ Cell adhesion, proliferation,	Yes	[120]
		↑ Cell adhesion, osteogenic differentiation, and bone growth in vivo	Yes	[121]
RGD + YGFGG (mammalian serum)	PET surfaces	↑ Osteogenic differentiation	—	[125]
			Yes	[126]
	Titanium surfaces	↑ Cell adhesion, osteogenic differentiation, osteogenesis, and osseointegration in vivo	—	[127]

**TABLE 4:** Combination of the RGD motif with BMP‐derived osteogenic peptides and major biological outcomes reported. The native GF mimicked by the peptide is indicated in brackets. The table also highlights when the geometrical disposition of the peptides was controlled.

Bioactive motifs	Substrate	Biological outcome	Geometry controlled	Refs.
RGD + KIPKASSVPTELSAISTLYL (BMP‐2)	PLEOF hydrogel	↑ Osteogenic differentiation	—	[129, 130]
	Titanium implants	↑ Cell adhesion, osteogenic differentiation, osseointegration; ↑ M2 polarization	—	[131]
	Glass substrate	↑ Osteogenic differentiation	—	[132, 133]
	Modular protein on TCPS	↓ Osteogenic differentiation	—	[134]
	PLGA‐PEG scaffold	↑ Cell adhesion, proliferation, and osteogenic differentiation	—	[135]
	Alginate hydrogel	↑ Smad signaling, osteogenic differentiation	—	[136]
RGD + KIPKASSVPTELSAISMLYL (BMP‐2)	Glass substrate	↑ Osteogenic differentiation	—	[137]
			Yes	[138]
	PET surfaces	↑ Osteogenic differentiation	—	[125, 139]
			Yes	[126]
		↑ Osteogenic differentiation, ECM thickness	—	[147]
RGD + DWIVA (BMP‐2) * In work [143] *cyclic variants of both RGD and DWIVA were used*	Alginate hydrogel	= Osteogenic differentiation (no synergy, no Smad signaling)	—	[136]
	Glass substrate	↑ Cell adhesion, p38 signaling; ↓C2C12 myogenesis	Yes	[142]
	Ti disks	↑ Cell adhesion, osteogenic genes, and bone formation	Yes	[143]
	Zirconia	↑ Cell adhesion, osteogenic differentiation	Yes	[144]
	PEG hydrogels	↑ Cell spreading, osteogenic differentiation	Yes	[145]
	Hyaluronic acid hydrogel	↑ Osteogenic differentiation, bone formation	—	[146]
RGD + RTVPKPSSAPTQLNAISTLYF (BMP‐7)	PET surface	↑ Osteogenic differentiation, ECM thickness	—	[147]
Cyclic RGD + GQGFSYPYKAVFSTQ (BMP‐7)	Glass substrate	↑ Cell adhesion, osteogenic differentiation	—	[148]
RGD + GQGFSYPYKAVFSTQ (BMP‐7)	Alginate hydrogel with silica NPs	↑ Cell proliferation, osteogenic differentiation	—	[149]
RGD + RKVGKASSVPTKLSPISILYK (BMP‐9)	PET surface	↑ Osteogenic differentiation, ECM thickness	—	[147]
KGGPQVTRGDVFTMP + KGGQGFSYPYKAVFSTQ (vitronectin + BMP‐7)	polydopamine/chitosan PS	↑ Osteogenic differentiation	—	[151, 152]
CGGNGEPRGDTYRAY + CGGKVGKACCVPTKLSPISVLYK/ CGGKVGKASSVPTKLSPISVLYK (BSP + BMP‐9)	PCL films	↑ MAPK and Runx2 signaling	—	[100]
CGGPHSRNGGGGGGRGDG + CGGKVGKACCVPTKLSPISVLYK/ CGGKVGKASSVPTKLSPISVLYK (FN + BMP‐9)				
YAVTGRGDSPASA + CGGKVGKACCVPTKLSPISVLYK + ACKIPKASSVPTELSAISTLYLA (FN + BMP‐9 + BMP‐2)	Silica films	↑ Osteogenic differentiation	—	[155]

### 5.1 Co‐Presentation of RGD With Other Cell Adhesive Peptides

A well‐described approach to selectively improve osteoblastic responses on biomaterials is to combine the integrin‐binding sequence RGD with peptides displaying affinity for cell surface heparan sulfate proteoglycans. One representative example is the peptide Phe‐His‐Arg‐Arg‐Ile‐Lys‐Ala (FHRRIKA), which is derived from the heparin‐binding domain of BSP. This protein is the major non‐collagenous protein in bone ECM and has osteoinductive properties with specificity toward osteoblast‐like cell attachment. Thus, in one of the first reports in this area, Rezania and Healy combined RGD and FHRRIKA on model quartz surfaces at different ratios. Although cell proliferation was found to be independent of the surface chemistry, the substrates containing the highest ratio of FHRRIKA better promoted cell spreading, and supported the highest strength of bone cell detachment. In addition, FHRRIKA‐rich surfaces enhanced mineralization, demonstrating a synergistic capacity of both peptides to induce osteogenic differentiation [104]. In a subsequent study, this approach was successfully transferred from 2D models to 3D materials, using NiPAM and acrylic acid (AAc) [poly(NiPAM‐co‐AAc)] hydrogels. The peptide‐modified hydrogels enhanced the spreading and proliferation of osteoblast‐like cells compared to non‐functionalized controls [105]. Since then, this strategy has been exploited by several other groups on different types of materials, including roughened titanium [106] or hydroxyapatite [107], although in the latter case, the peptide coatings failed to significantly improve cell adhesion compared to RGD alone, and did not reach the adhesion levels observed on serum‐coated hydroxyapatite. Another limitation of the mentioned works is that the geometrical presentation of the different peptides was not chemically controlled, and hence, the adequate spacing and presentation of the peptides to engage in synergistic signaling was not investigated. A remarkable example that could overcome this was reported in the work by Pagel et al. In this study, the authors introduced a novel functionalization approach combining cyclic RGD and FHRRIKA within a single molecule. This was achieved by using Diels–Alder and azide–alkyne click conjugation reactions, and allowed controlling the exact ratio of each peptide present on the titanium surface and the spacing between them [108]. This approach resulted in enhanced osteoblastic responses in a cooperative manner, compared to the presentation of the individual peptides or mixtures without spatial control. However, despite these promising outcomes, the potential of this strategy to promote bone regeneration in vivo was not explored.

Another heparin‐binding motif that has been widely studied is the Lys–Arg–Ser–Arg (KRSR) sequence. This peptide was designed following a consensus pattern found in five different bone‐related adhesive proteins (FN, VN, BSP, thrombospondin, and osteopontin (OPN)), and is known to specifically promote osteoblast adhesion and osteogenic differentiation [51, 106, 109]. Such selectivity toward bone‐forming cells was demonstrated in a seminal contribution by Hasenbein et al., in which this peptide was shown to foster the adhesion of osteoblasts but not of fibroblasts [109]. Based on these evidences, further works employed the KRSR sequence together with RGD aiming at synergistically enhancing osteoblast‐like activity. However, conflicting results were reported in the literature, and while some studies found positive cooperative effects of the two peptides on osteoblastic responses [106, 110], others concluded that there were no statistically significant differences in comparison to the use of the individual peptides alone [107, 111], or even reported negative results. For example, Bell et al. described that the co‐presentation of RGD and KRSR had an inhibitory effect on osteoblast differentiation [112]. The same strategy, applied to titanium implants, failed to enhance bone‐implant contact (BIC), bone fill, or interfacial shear strength in an in vivo study, compared to the non‐functionalized implants [113]. These findings led the authors to conclude that, in addition to optimizing the peptide density and mixture ratios, the spatial distribution of the peptides on the surfaces required further investigation. In response to these shortcomings, we engineered a dual molecular‐based platform, in which the exact amount and molar ratio (1:1) of RGD and KRSR were chemically controlled. This allowed the creation of a dual‐peptide biointerface that was covalently grafted to titanium via silanization [114]. Cellular assays with osteoblastic Saos‐2 cells demonstrated the capacity of the biointerface to remarkably foster cell adhesion, projected area, and proliferation compared to bare titanium. In addition, a synergistic effect on increasing cell mineralization was observed in comparison with the presentation of the peptides alone, further confirming the relevance of integrating bioactive cues in a geometrically controlled manner to elicit favorable receptor signaling.

Another relevant example is the sequence Pro‐His‐Ser‐Arg‐Asn (PHSRN). As previously described in Section 3, this peptide is found in the ninth type III repeat of FN (i.e., FN III9) and synergistically enhances the binding of RGD to α_5_β_1_ in FN [115]. To this purpose, Kim et al. developed oligopeptides made of RGD and PHSRN sequences. Of interest, the two motifs were separated using Gly spacers of different lengths. They showed an enhancement in cell adhesion and MAPK signaling, which was dependent on both the concentration of the coating and the spacing between the peptides [116]. Following a similar approach, Benoit and Anseth produced poly(ethylene glycol) (PEG) hydrogels functionalized with RGD and PHSRN, which were spaced by a Gly_13_ chain that mimics the ∼35–40 Å distance present in the native conformation of FN. The functionalized hydrogels statistically increased the number of adhering osteoblasts, their projected area and focal adhesions, as well as their metabolic activity and ALP production, compared to non‐functionalized controls and RGD alone [117]. Alternatively, these two sequences were also immobilized on alginate hydrogels. However, in this case, instead of combining both motifs in the same polymeric chain, RGD‐modified alginate and PHSRN‐modified alginate were mixed at different ratios and crosslinked. Alginate hydrogels with a concentration of RGD higher than 33% enhanced cell attachment and differentiation [118]. Likewise, mixed solutions of RGD and PHSRN peptides were also used to coat titanium disks. These dual‐functionalized surfaces also showed improvement of cell adhesion and ALP activity compared to mono‐functionalized controls [119]. Collectively, these previous works illustrate the potential of co‐presenting RGD and PHSRN to promote α_5_β_1_‐dependent cell adhesion and differentiation, but also stress the importance of controlling peptide density, ratio, and geometrical disposition to mimic the cell adhesion site of FN, which encompasses FN III9 (PHSRN) and FN III10 (RGD) at a defined distance. In line with this, we designed a molecular peptidic platform tethering the two sequences mimicking their native spacing in FN (i.e., the 35–40 Å separation). A crucial feature in the design was also the incorporation of anchoring groups to allow for binding to the surface, without interfering with the biological activity, and a branching unit to provide an optimal accessibility (open conformation) of the bioactive peptides. This strategy demonstrated to be effective in synergistically boosting cell adhesion and proliferation of osteoblasts [120], as well as the osteogenic differentiation of MSCs, evidenced by increased levels of ALP activity, osteogenic gene expression, and mineralization [121]. Of note, such responses were enhanced in comparison not only to the peptides RGD and PHSRN alone, but also when they were presented as a mixture, in which the geometrical disposition of the peptides was not controlled, and thus the native spacing present in FN was not matched. Furthermore, the multi‐peptidic biointerface also promoted higher rates of bone formation in vivo [121].

A fourth example is represented by the 14‐mer osteogenic growth peptide (OGP), an endogenous regulatory peptide present in mammalian serum that is identical to the C‐terminus of histone H4 and stimulates osteoblastic activity [122]. In particular, the active portion capable of enhancing the proliferation and differentiation of osteoblasts was found to be located at the C‐terminal fragment spanning the sequence Tyr‐Gly‐Phe‐Gly‐Gly (YGFGG) [123, 124]. Based on these evidences, Durrieu and co‐workers functionalized polyethylene terephthalate (PET) model surfaces with combinations of different peptides, including RGD and YGFGG. An increased expression of both bone‐related genes and proteins confirmed the capacity of the surfaces to promote MSCs osteogenic differentiation [125]. In a more recent study, the same research group further investigated the influence of controlling the spatial organization of both peptides by using diverse geometrical patterns. They found that MSCs better switched into osteoblast cells in relatively small patterns (area smaller than 625 µm^2^) and with sharp shapes, like rectangles [126]. These results are meaningful, as bigger areas would not allow an adequate co‐presentation of the peptidic molecules and the corresponding activation of cell‐expressed receptors. Another reported strategy focused on the use of mussel‐derived l‐3,4‐dihydroxyphenylalanine (DOPA) molecules to bind mixtures of RGD and YGFGG at different ratios on titanium surfaces. Although no statistically significant differences were observed in cell adhesion across the different conditions, MSCs osteogenic differentiation was significantly improved when using RGD and YGFGG at a 1:3 ratio. In vivo experiments demonstrated that titanium screws functionalized with such a peptide combination enhanced osteogenesis, BIC, and mechanical stability of the implants [127]. Very recently, Shou et al. also engineered RGD and YGFGG sequences incorporating a short poly‐l‐lysine chain (i.e., Lys_6_), which was used as an assembly unit to assist the binding of the peptides to metal polyphenol networks by supramolecular interactions. These coatings were able to promote osteogenic differentiation and inhibit osteoclast differentiation in vitro, while enhancing bone regeneration and osseointegration in vivo [128].

### 5.2 Co‐Presentation of RGD With BMP‐Derived Osteogenic Peptides

As previously explained, among the different types of BMPs that have been identified to date, BMP‐2 stands out for its capacity to induce bone formation, being the only one—together with BMP‐7—to receive regulatory approval and reach clinical practice. Thus, an extensive amount of research has been devoted to identifying and developing peptides that recapitulate the osteogenic domains of BMP‐2, without exerting its well‐documented side effects (Table 4). Moreover, the combination of BMP‐derived peptides with RGD has the potential to engage in synergistic integrin‐GF signaling (see Section 2.3). In this regard, one of the most well‐studied motifs has been the sequence KIPKASSVPTELSAISTLYL (and its variant with the mutation T89M), which corresponds to the residues 73–92 of the knuckle epitope of BMP‐2. Indeed, earlier studies conducted by He et al. showed the osteogenic capacity of this peptide co‐presented with RGD. The authors used a poly(lactide‐co‐ethylene oxide‐co‐fumarate) (PLEOF) macromere as a substrate, which was crosslinked with RGD and propargyl acrylate to produce an RGD‐modified hydrogel. Afterward, the BMP‐2 peptide was grafted to the hydrogel by click chemistry, reaching a final ratio of about 1:3 (RGD:BMP‐2). Although statistically significant differences were not observed within the peptide‐functionalized conditions, the co‐presentation of the two motifs clearly induced the switch of MSCs into osteoblasts, as highlighted by significant increases in ALP activity, mineralization, and overexpression of osteogenic genes, compared to RGD or the BMP‐2 peptide alone [129, 130]. In line with these positive outcomes, the same proportion of the two peptides (1:3) was recently used to functionalize titanium implants by means of catechol‐assisted chemistry. Notably, the biofunctionalized implants not only promoted osseointegration in vivo, but were also able to regulate the polarization of macrophages to the M2 phenotype and inhibit inflammation [131]. The Becker's research group also combined both sequences on glass substrates, but employing gradient concentrations. They observed an increased osteogenic differentiation for the combined peptides, and reported a threshold total concentration of 130 pmol/cm^2^ (65 pmol/cm^2^ of each RGD and BMP‐2‐peptide) required to synergistically upregulate the expression of BSP and the promotion of mineralization, without osteogenic supplements [132]. In a subsequent study, the same group presented a more sophisticated method to prepare orthogonal concentration gradients of the two peptides, better controlling their presentation ratio, aiming at deciphering the synergistic mechanism of RGD in combination with the BMP‐2‐peptide. The authors found accelerated MSC differentiation using RGD and BMP‐2 at 71−83 and 25 pmol/cm^2^, respectively, compared to the individual peptide concentrations. However, the same effects could not be reproduced when presenting different concentrations/ratios of the peptides, corroborating the strong dependence on the concentration and spatiotemporal features of the tethered peptides to replicate the complex crosstalk between integrin‐GF intracellular pathways [133]. On the contrary, other works could not demonstrate a synergistic signaling between these two peptides. For instance, Kim et al. produced modular proteins containing either the RGD or the BMP‐2 motif by recombinant methods, which were used to coat tissue culture polystyrene (TCPS) individually or as a mixture. However, in this case, the co‐presentation of the two motifs did not have a positive effect on osteodifferentiation and even inhibited it in comparison with the use of the BMP‐modified protein alone [134]. These results could be due to differences in the respective peptide concentration, which, as discussed in the previous works, is a crucial aspect to ensure effective signaling. The authors also hypothesized that the RGD‐containing protein could preferentially bind α_v_β_3_ and that activating this integrin may suppress osteogenic differentiation; yet this was not investigated in their study, and the precise role of α_v_β_3_ in osteodifferentiation remains controversial. Moreover, another plausible explanation for the observed effects could be that the active sequences were not accessible enough for co‐stimulating integrins and BMP receptors in the context of the conformation adopted by the modular proteins. Contrary to this, the co‐immobilization of the two motifs together with hydroxyapatite nanoparticles on PLGA‐PEG copolymers significantly enhanced MSCs adhesion, proliferation, and osteodifferentiation [135]. Furthermore, the combination of the two peptides on alginate hydrogels was shown to increase ALP activity and mineralization compared to RGD, an effect likely due to the capacity of the BMP‐2 peptide to initiate Smad signaling [136].

**FIGURE 7:**
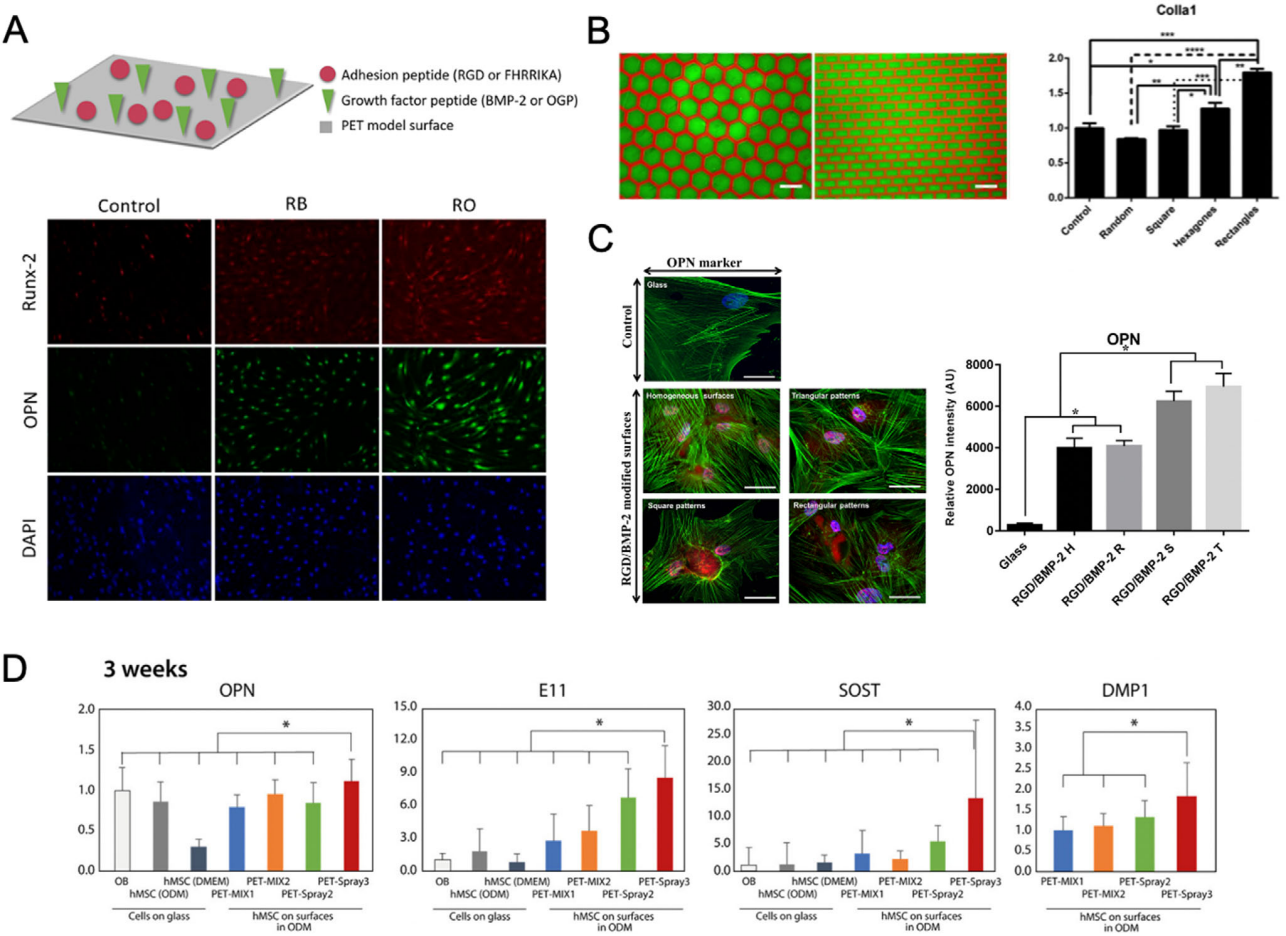
Co‐immobilization of RGD with a BMP‐2‐derived peptide. (A) Grafting of peptide mixtures on PET surfaces: RGD and BMP‐2‐derived peptide (RB) or RGD and OGP peptide (RO). The peptide mixtures increase Runx‐2 and OPN staining, compared to non‐modified surfaces. Reprinted (adapted) with permission from [125]. Copyright 2018 American Chemical Society. (B) Representative micropatterns coated with the peptides and Colla1 expression as a function of the geometries. Reprinted (adapted) with permission from [126] Copyright 2020 John Wiley and Sons. (C) Staining of the late osteogenic marker OPN and quantity analysis, showing different expression depending on the pattern's shape. Reprinted (adapted) with permission from [138]. Copyright 2017 American Chemical Society. (D) A spray technology was used to randomly micropattern RGD and BMP‐2‐derived peptides on PET surfaces. Results show the expression of osteoblast and osteocyte markers comparing a disordered versus a homogenous peptide presentation. Reprinted (adapted) with permission from [139]. Copyright 2023 Royal Society of Chemistry.

Alternatively, other authors have studied a variant of the knuckle‐derived BMP‐2 peptide, the sequence KIPKASSVPTELSAISMLYL, in which a threonine residue is mutated to methionine (T→M) and combined it with RGD (Figure 7). In this regard, Durrieu and coworkers demonstrated the capacity of the two motifs to synergistically enhance MSCs differentiation on glass substrates, as highlighted by a significantly increased expression of the osteogenic marker Runx2 [137]. Following a similar approach, the same research group functionalized PET surfaces with a mixture of the two peptides (Figure 7A), observing increased staining of Runx‐2 and OPN, and higher expression of ALP and Col‐IA genes, compared to non‐functionalized controls [125]. In subsequent studies, the spatial distribution of the dually grafted peptides was fine‐tuned with different micropatterned geometries (Figure 7B). Notably, such geometrical cues significantly affected the osteogenic differentiation of MSCs, with particular shapes inducing the overexpression of Runx2, Col‐IA, and OPN genes and proteins (Figure 7C), as well as ALP activity, highlighting a strong interplay between topographical and biochemical signals to foster osteodifferentiation [126, 138]. In a recent study, the authors used a spray technology to micropattern both peptides on PET surfaces; interestingly, they showed that the disordered micropatterned surfaces with RGD and BMP‐2 promoted higher MSCs osteodifferentiation than the surfaces displaying a homogeneous distribution of the two peptides [139] (Figure 7D).

**FIGURE 8:**
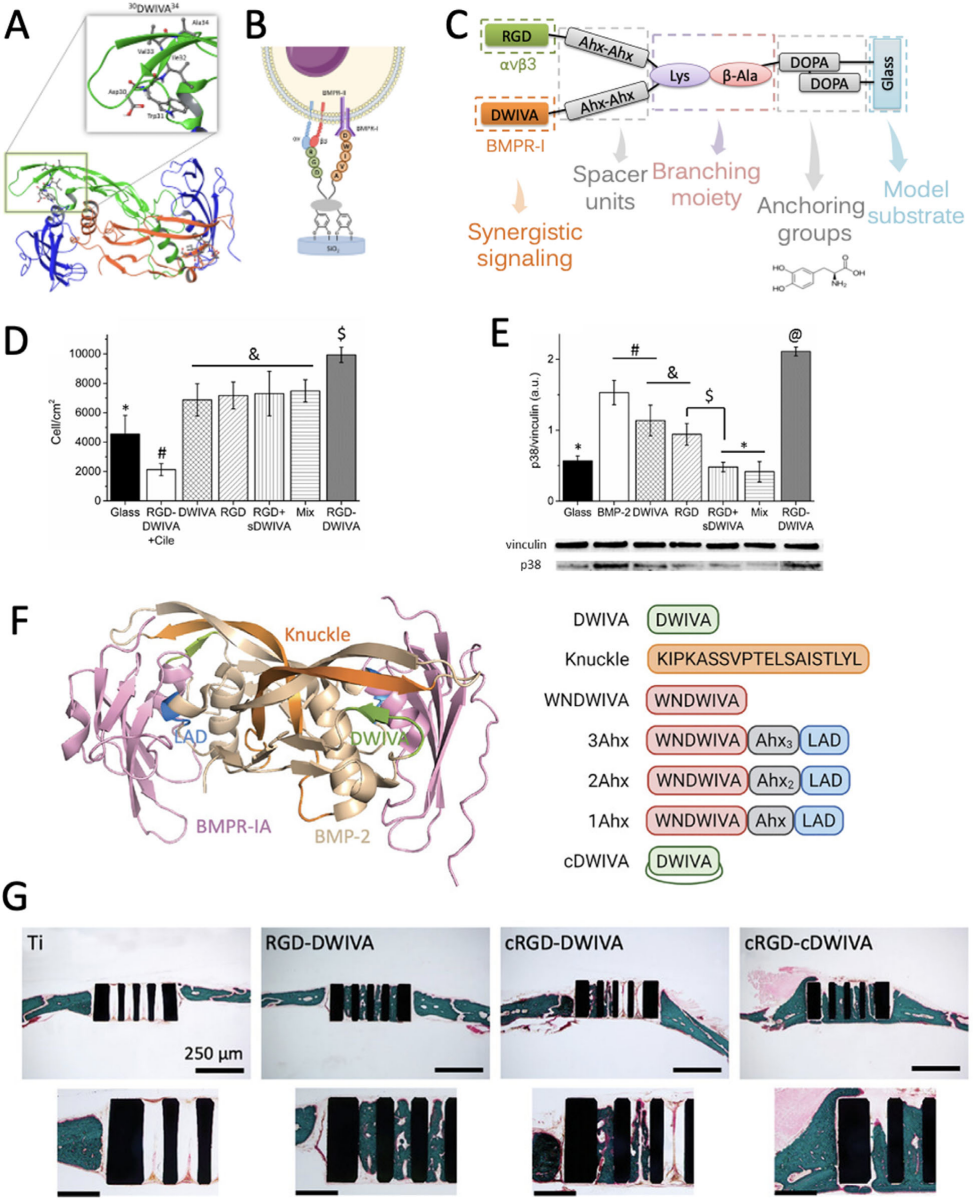
Co‐immobilization of RGD with the DWIVA peptide. (A) The DWIVA sequence covers residues 30–34 of the wrist epitope of BMP‐2; it is involved in BMP‐2‐BMPR‐I interactions. Reprinted (adapted) with permission from [142] Copyright 2021 John Wiley and Sons. (B) The co‐presentation of RGD and DWIVA may engage in synergistic signaling by co‐activating integrins and GFRs, respectively. Reprinted (adapted) with permission from [142] Copyright 2021 John Wiley and Sons. (C) Schematic representation of the multifunctional peptidic platform integrating RGD and SWIVA. The key structural and functional elements of the molecule are highlighted. (D) Synergistic improvement of C2C12 adhesion by the RGD‐DWIVA biointerfaces. Reprinted (adapted) with permission from [142] Copyright 2021 John Wiley and Sons. (E) BMP‐signaling is mediated by p38 following a Smad‐independent pathway. Reprinted (adapted) with permission from [142] Copyright 2021 John Wiley and Sons. (F) Potential osteogenic sequences identified in BMPR‐IA‐binding domains in BMP‐2, especially in the wrist region. Reproduced from [143] (Creative Commons CC BY license). (G) The functionalization of titanium implants with RGD‐DWIVA peptides significantly enhanced in vivo bone formation compared to non‐functionalized implants. Reproduced from [143] (Creative Commons CC BY license).

Another binding epitope that has been identified in BMP‐2 is the wrist epitope, which binds to BMP receptor type I (BMPR‐I) with high affinity [46, 140]. In this regard, the sequence Asp‐Trp‐Ile‐Val‐Ala (DWIVA), spanning residues 30–34 of the wrist region, has been described to be mostly involved BMP‐2‐BMPR‐I interactions [140] and to have osteoinductive capacity (Figure 8A) [141]. Nonetheless, one of the first attempts to combine RGD and DWIVA on alginate hydrogels failed to prove a positive crosstalk on cell adhesion, signaling, and osteodifferentiation [136]. The strategy employed, though, did not effectively control the distance or presentation between the two peptides. Indeed, we recently demonstrated that the DWIVA motif requires the co‐presentation with RGD in a geometrically controlled manner to promote cell adhesive events (Figure 8B), as the proximity for engagement of integrins and BMPRs at the ventral side of the cell is required to activate synergistic integrin and GF signaling [142]. To this end, we developed branched peptidic platforms that were employed to integrate the two peptides, fine‐tuning their spacing and accessibility for receptor interaction, and immobilized these molecules on model glass substrates (Figure 8C). Of note, the RGD‐DWIVA biointerfaces synergistically improved C2C12 adhesion, inhibited myoblast differentiation, and activated p38 expression in comparison to the individual peptides, their random distribution as mixtures, or the presentation of immobilized RGD and soluble DWIVA (Figure 8D,E), thus stressing the importance of the spatial control of the peptides for synergistic signaling. It is also relevant to note that the signaling was found to be α_v_β_3_ dependent and Smad independent—which could also explain why Smad‐dependent activation was previously not detected [136]. In a subsequent study, we designed a series of synthetic peptides screening BMPR‐IA‐binding domains in BMP‐2, aiming at identifying new osteogenic sequences. The well‐reported knuckle‐derived sequence was included as a control, and wrist‐derived peptides containing the DWIVA and LAD motif were studied (Figure 8F) [143]. To our surprise, only the DWIVA peptide (and its cyclic analogue) was able to significantly inhibit myotube formation on C2C12 cells, indicating that the DWIVA sequence seems to be structurally optimized in its native structure, as even subtle changes result in loss of activity. Moreover, the knuckle epitope was inactive in the C2C12 model. Such a lack of activity, compared to the reported studies previously discussed, may result from differences in the cellular models used, peptide concentration, ratio, and presentation modes. Further studies comparing both the knuckle and wrist epitopes are warranted. Next, analogues containing RGD and DWIVA (linear or cyclic combinations) were used to functionalize titanium and fostered significantly higher MSC adhesion, focal adhesion formation, mineralization, ALP activity, and osteogenic gene expression, compared to RGD or DWIVA alone. Furthermore, implantation of titanium implants coated with the dual peptides in rat calvarial defects enhanced in vivo bone formation (Figure 8G) [143]. The RGD‐DWIVA platform has been applied to other materials, including micropatterned zirconia [144] and protease‐degradable PEG hydrogels [145]. In both cases, the presence of the peptides significantly improved MSC adhesion and spreading and upregulated the expression of osteogenic genes, evidencing the capacity to promote osteodifferentiation of this strategy. Similarly, Gultian et al. developed injectable hyaluronic acid hydrogels with immobilized RGD and DWIVA, which showed the capacity to promote osteogenic differentiation in vitro as well as regeneration of trabecular bone in vivo [146].

In addition to the knuckle and wrist epitopes of BMP‐2, other fragments of BMPs with relevant roles in bone formation have been investigated. This is the case of the sequences corresponding to residues 89–117 (RTVPKPSSAPTQLNAISTLYF) of BMP‐7 and 68–87 (RKVGKASSVPTKLSPISILYK) of BMP‐9. These peptides and the knuckle BMP‐2 peptide were combined with RGD, respectively, and immobilized on PET surfaces. Interestingly, the three sequences significantly enhanced the osteogenic differentiation of pre‐osteoblastic cells, as indicated by a higher expression of characteristic osteogenic genes, increased mineralization, changes in cell morphology and ECM production, compared to RGD. Within the three peptides, the highest effects were observed for the BMP‐2 peptide, followed by the BMP‐7 and the BMP‐9 derivatives [147]. Another example is the short bone‐forming peptide (BFP‐1, sequence: GQGFSYPYKAVFSTQ), which is found in the immature region of BMP‐7 and has been reported to have higher osteogenic potential than the original protein. Indeed, the co‐immobilization of BPF‐1 with RGD on both 2D and 3D substrates fostered increased MSC adhesion, proliferation, and osteogenic differentiation [148, 149, 150].

Finally, diverse BMP‐derived peptides have also been combined with RGD within larger peptidic sequences, normally to cover a wider range of the native ECM protein or to include multiple signaling. For example, the cell‐adherent KGGPQVTRGDVFTMP sequence, derived from VN, was combined with BFP‐1 to functionalize polydopamine/chitosan‐coated polystyrene (PS). The biofunctional substrates induced the switch of human pluripotent stem cells (PSCs) into osteoblasts, as shown by gene expression, ALP activity, and mineralization [151, 152]. Interestingly, the VN peptide alone was also able to direct the differentiation of PSCs to the osteoblastic phenotype when grafted on PCL fibers [153]. Also employing PCL substrates as material, we previously reported in Section 4 (Table 2) the notable work of Beauvais et al. in which a BSP (CGGNGEPRGDTYRAY) and a FN‐derived peptide (CGGPHSRNGGGGGGRGDG) were combined with BMP‐9 [100]. In that study, the authors also investigated two BMP‐9 peptides, the sequence CGGKVGKACCVPTKLSPISVLYK, derived from residues 68–87 of the knuckle epitope of BMP‐9, and its variant CGGKVGKASSVPTKLSPISVLYK (in which cysteine residues 76 and 77 are replaced by two serine). FN‐modified PCL together with BMP‐9‐derived peptides induced higher levels of Smad phosphorylation and Runx2 expression in comparison with PCL films functionalized with the BSP peptide and BMP‐9‐derived motifs. However, this last condition also improved the phosphorylation of Smad and Runx2 signaling in regard to controls without the stimulation of BMP‐9 peptides [100]. More recently, the same research group demonstrated that the combination of the FN peptide with the BMP‐9‐derived peptide increased ALP activity and Smad signaling, and also upregulated the expression of Osterix and ALP genes of preosteoblastic cells [154]. Silica films were also functionalized with another FN‐derived sequence (YAVTGRGDSPASA) together with the previously mentioned knuckle‐derived peptide CGGKVGKACCVPTKLSPISVLYK (from BMP‐9) or with the BMP‐9 and also the knuckle‐based ACKIPKASSVPTELSAISTLYLA (from BMP‐2). In both cases, the biointerfaces slightly enhanced the differentiation of pre‐osteoblastic cells [155].

## 6 Co‐Presentation of Other Cell Adhesive Sequences With BMP‐Derived Peptides

This review has primarily focused on the use of RGD as the major integrin‐binding peptide responsible for cell attachment to ECM proteins. However, a significant number of other integrin‐binding peptides or sequences with affinity for other receptors have been described, and can potentially be associated with BMP‐derived molecules for improving osteodifferentiation and bone formation (Table 5). One representative example is the triple helical, α_2_β_1_‐selective GFOGER sequence present in collagen [39]. This peptide was used by García and coworkers to functionalize protease‐degradable PEG hydrogels, which were used as BMP‐2 delivery vehicles. Of note, GFOGER‐functionalized hydrogels (without BMP‐2) were effective in directing osteodifferentiation and bone formation in critical‐sized defects in mice compared to RGD‐functionalized hydrogels. Moreover, the incorporation of BMP‐2 allowed for a dose‐dependent healing response, achieving enhanced bone repair at very low BMP‐2 doses. Of note, the same concentration of BMP‐2 failed to promote bone regeneration when delivered from collagen sponges, the current clinical carrier. These findings point toward a synergistic crosstalk between α_2_β_1_ integrin and BMP receptors [156].

**TABLE 5:** Combination of the non‐RGD sequences with BMP‐derived osteogenic peptides. The native proteins (or sources) for each sequence are indicated in brackets.

Bioactive motifs	Substrate	Biological outcome	Refs.
GFOGER + BMP‐2 (collagen + BMP‐2)	PEG hydrogel	↑ Osteogenic differentiation, bone regeneration in vivo	[156]
FHRRIKA + YGFGG (BSP + mammalian serum)	PET surface	↑ Osteogenic differentiation	[125]
FHRRIKA + KIPKASSVPTELSAISMLYL (BSP + BMP‐2)			
KRSR + FHRRIKA + NSPVNSKIPKACCVPTELSAI (fibronectin + BMP‐2)	PLGA + hydroxyapatite	↑ Osteogenic differentiation, bone formation in vivo	[157]
KIPKASSVPTELSAISTLYL‐AAAA‐γEPRRγEVAγEL (BMP‐2 + osteocalcin)	Hydroxyapatite coating on PLGA film	↑ Osteogenic differentiation	[158]
EPLQLKM + KIPKACCVPTELSAISMLYL (phage display + BMP‐2)	Electrospun PCL	↑ Cell adhesion, osteogenic differentiation	[160]
(GSGAGA)_n_ + GQGFSYPYKAVFSTQ (silk fibroin + BMP‐7)	Calcium titanate nanorods	↑ mineralization; (↓ osteogenic gene expression)	[161]
KPSSAPTQLN and/or SNVILKKYRN and/or KAISVLYFDDS (BMP‐7)	In solution	↑ Cell proliferation, mineralization	[162]
KIPKASSVPTELSAISMLYL‐GPGG‐ DWIVA (BMP‐2)	TCPS	↑ Cell adhesion, osteogenic differentiation	[163]

Heparin‐binding peptides, which have been used in cooperation with RGD (Section 5.1, Table 3), can also be combined with BMP‐derived peptides. For example, FHRRIKA was co‐presented on PET surfaces with either YGFGG (OGP) or with the knuckle epitope of BMP‐2. In both cases, overexpression of Runx2, Col‐IA, and ALP genes confirmed their ability to induce osteogenic differentiation [125]. FHRRIKA was also used together with the heparin‐binding sequence KRSR and a knuckle‐derived peptide from BMP‐2 (SPVNSKIPKACCVPTELSAI) to functionalize porous membranes made of PLGA and hydroxyapatite layers. The co‐immobilization of the three peptides aimed at improving MSC adhesion, proliferation, and osteoblastic differentiation. Indeed, an increase in ALP activity, as well as in OCN and OPN protein expression, corroborated the effectiveness of the surface functionalization in enhancing osteodifferentiation. Moreover, implantation of such membranes on a non‐healing rat calvarial defect promoted new bone formation [157].

OCN is another protein found in bone ECM, which is also involved in cell adhesion and osteogenesis. Taking advantage of the hydroxyapatite‐binding region present in its N‐terminal region (γEPRRγEVAγEL; γE = γ‐carboxylated glutamic acid), Murphy's research group combined this sequence with the BMP‐2 knuckle sequence in a modular fusion peptide and decorated PLGA films coated with hydroxyapatite. Overexpression of BMP‐2 and OCN, as well as higher levels of ALP activity and mineralization, compared to controls, demonstrated the osteogenic capacity of such biointerfaces [158]. Although in this case the two peptides were not used for their potential synergistic signaling, this work represents a good example of how a modular peptidic design can be used to incorporate substrate‐specific anchoring units, based on ECM native interactions. The knuckle‐derived peptide from BMP‐2 was also recently combined with the heptapeptide sequence EPLQLKM (named as E7), which had been discovered by phage display and has high affinity for MSCs [159]; in particular, electrospun PCL scaffolds containing both peptides simultaneously promoted MSCs adhesion and osteogenic differentiation [160].

Following a different approach, Sun et al. coated calcium titanate nanorods with fibroin, which was used as a cell adhesive matrix, and the BMP‐7‐derived BFP‐1, aiming at improving osteoblast differentiation. However, although a minor increase in mineralization was observed, the fibroin‐BFP‐1 biointerface seemed to reduce the expression of Runx2 and Col1 genes, contrary to the authors’ claims, making it difficult to assess clear synergistic events [161].

Finally, an alternative strategy has focused on combining peptides derived from different regions of BMPs, without using cell‐adhesive sequences. For example, Chen and Webster tested the effects of combining three peptides derived from distinct regions of BMP‐7 (KPSSAPTQLN, SNVILKKYRN, and KAISVLYFDDS) on osteoblastic activity, reporting increased proliferation and calcium deposition for specific combinations [162]. Similarly, the two osteogenic motifs derived from the knuckle and wrist epitopes of BMP‐2 were combined using a chimera peptide (KIPKASSVPTELSAISMLYL‐GPGG‐DWIVA). In solution, this peptide was found to foster cell adhesion and osteodifferentiation [163].

## 7 Conclusions and Perspectives

Bone ECM provides a rich microenvironment encompassing a large number of signaling cues that regulate the physiology of bone remodeling and healing. Mimicking such signaling on biomaterials is thus a powerful strategy to fine‐tune cell adhesion, promote osteodifferentiation, and ultimately drive bone tissue regeneration. Since the discovery of the RGD motif in 1984 as the minimal cell‐binding sequence of FN, reproducing integrin signaling has been a major goal; however, now we know that ECM‐integrin interactions recapitulate only a small fraction of the bone healing microenvironment, and that signaling through other receptor pathways is needed.

To address this complexity, researchers have increasingly turned to BMPs, a group of GFs known for their potent osteoinductive capabilities. BMPs not only play critical roles in directing stem cell fate, enhancing matrix production, and promoting vascularization, but also complement integrin‐mediated adhesion by activating distinct signaling pathways and engaging in synergistic signaling. Incorporating BMP signaling into biomaterial design, therefore, represents a more holistic approach to replicating the multifaceted signaling environment of native bone ECM, with the potential to improve outcomes in bone tissue engineering. This review focuses on representative strategies developed to achieve this goal.

A major landmark in this area of research has been the discovery that BMP activity can be regulated and enhanced by integrin signaling. In line with this, substantial research has investigated the combination of ECM proteins with BMPs. The most representative example is FN, which encompasses both a cell attachment site and a promiscuous GF‐binding domain. Notably, this strategy accurately reproduces the natural environment of bone and has been shown in several animal models to promote synergistic osteogenic signaling and new bone formation, using GF doses significantly lower than those generally used in clinical practice. These findings hold great potential for clinical translation and could overcome the safety concerns associated with GF‐based therapies.

Taking advantage of the ability to reproduce specific biological functions of proteins using synthetic peptides, an increasing number of studies have aimed to replace full‐length ECM proteins with shorter sequences. The canonical example is RGD, which mimics the cell‐attachment site of many ECM proteins. This approach has also been extended to several BMPs, with the goal of identifying osteogenic domains capable of reproducing the GF's activity while minimizing undesired side effects. A key advantage of using peptides is their amenability to precise integration on biomaterial surfaces, offering greater control over substrate specificity, coating density, and spatial presentation—parameters that are generally difficult to regulate with native proteins.

A well‐studied GF in this context is BMP‐2, for which at least two regions have been identified to be involved in receptor binding and osteogenic signaling: the “knuckle” epitope (encompassing the sequence KIPKASSVPTELSAISTLYL) and the “wrist” epitope (containing the sequence DWIVA). These peptides have been used together with RGD by several research groups to elicit synergistic signaling. Overall, most studies have reported positive (cooperative) effects from the combination of RGD with BMP‐2‐derived peptides. However, the achievement of true synergistic signaling strongly depends on multiple factors, including peptide concentration, ratio, accessibility, and spatial distribution. Given that different studies employ diverse strategies of functionalization (e.g., peptide mixtures, fusion peptides, branched architectures), the reported effects are not always directly comparable. More systematic investigations are required to elucidate the optimal conditions for effective integrin‐GFR crosstalk.

For instance, the BMP‐2 knuckle‐derived peptide was initially described to best exert its osteogenic potential when combined with RGD in a final 1:3 ratio (RGD:BMP‐2) (1.62 and 5.2 pmol/cm^2^, respectively) [129]. This same ratio showed enhanced osseointegration of biofunctionalized titanium implants [131]. In contrast, other authors reported increased osteogenic differentiation using an equimolar 1:1 ratio of the same peptides (RGD:BMP‐2) (65 pmol/cm^2^ of each peptide)[132] or even using higher concentrations of RGD, i.e., a 3:1 ratio (RGD:BMP‐2) (71–83 pmol/cm^2^ and 25 pmol/cm^2^, respectively) [133]. In addition, Durrieu and coworkers found a synergistic enhancement of MSC differentiation when co‐presenting a variant of the knuckle peptide with RGD in a ≈1:1.5 ratio (RGD:BMP‐2) (70 and 100 pmol/cm^2^, respectively) [137]. As mentioned above, these discrepancies may arise from the different strategies of immobilization and surfaces used, as well as from the different methods of physicochemical characterization. Another reason is that controlling the exact binding ratio on a surface is not always possible, as even if the same chemistry of conjugation is used for both peptides, differences in size, charge, and other physiochemical properties may affect the peptide's individual grafting ratio. A possible solution was introduced by us with the use of synthetic molecules that present both sequences within the same peptidic architecture in a chemically defined 1:1 ratio. This strategy was successfully applied to the co‐presentation of RGD and the BMP‐2 wrist‐derived DWIVA sequence, yielding a density of 38.4 pmol/cm^2^ on glass and 77.4 pmol/cm^2^ on titanium of each peptide, and showing synergistic integrin‐GF signaling and increased bone growth in vivo [142, 143]. Yet, this strategy is also limited because it only enables the study of equimolar concentrations, preventing the exploration of other ratios, such as 1:3 or 3:1, that may be biologically relevant, as demonstrated for the knuckle peptide. In terms of peptide concentration, the literature's reports also differ, but it seems that although synergistic signaling has been observed with concentrations as low as 1–5 pmol/cm^2^, the majority of studies point to concentrations above 25–50 pmol/cm^2^ to achieve optimal effects.

Another critical aspect to take into account is the spatial arrangement and presentation of integrin and GFR‐binding ligands to engage in effective signaling. Indeed, in a seminal contribution Cavalcanti‐Adam and colleagues demonstrated that BMP‐2 in its immobilized form (i.e., presented at the ventral side of the cell) was more efficient than in its soluble form (i.e., presented at the dorsal side of the cell) to stimulate the activation of the Smad‐transcriptional pathway,[95] as well as increasing integrin‐mediated cell spreading, focal adhesion assembly and downstream signaling [96]. These findings should not come as a surprise, as they reproduce the physiological capacity of BMP‐2 to interact with ECM proteins, such as FN, which also present integrin binding sites to stimulate cell adhesion and differentiation (see Section 3 of this review). Indeed, the Picart research group and others have reported similar findings [90, 164, 165]. Of interest, we were also able to reproduce these results with synthetic peptides. More specifically, we showed that the co‐immobilization of RGD and DWIVA peptides on glass model surfaces synergistically activated BMP‐dependent signaling via p38; in contrast, the presentation of immobilized RGD and soluble DWIVA (dorsal interaction) failed to produce the same biological effects, thus underscoring the necessity of presenting the ligands at the ventral side of the cell (i.e., in a matrix‐bound configuration) [142].

Less clear, however, is which geometrical orientation is optimal for activating synergistic integrin and GF signaling with peptides. Based on the existing literature, it seems evident that the ligands need to be presented in close vicinity to enable integrin and BMPR interactions and receptor crosstalk. It is also known that some degree of geometrical control over the distance and presentation of the peptides is needed, as the lack of such control has been associated with loss of activity [136, 142]. Systematic studies exploring this aspect are therefore warranted, as they could potentially provide very helpful information on the structural requirements for synergistic signaling.

Furthermore, the use of short synthetic peptides is not exempt from limitations. In the first place, unless adequately chemically modified, they are susceptible to enzymatic degradation by serum proteases, clearly limiting their use in vivo. In addition, synthetic peptides generally fail to mimic the specificity and complexity exhibited by ECM proteins, in which the binding epitopes are exposed in a biologically optimal conformation. In fact, linear peptides are flexible and can adopt multiple conformations, thus docking into structurally related receptors with similar binding modes, decreasing their selectivity, e.g., capacity to discriminate between different integrin subtypes. Moreover, proteins are multifunctional by nature and encompass in their sequence complementary or synergistic domains, which better recapitulate the healing microenvironment of the ECM (see Section 3 of this review). In contrast, synthetic peptides lack such additional functional sites and therefore cannot exert multiple interactions, which are in many instances required to trigger specific cell responses [41, 65, 102, 103].

These limitations have been partially addressed for RGD‐based integrin binding peptides by means of conformational restriction by cyclization, site‐specific chemical modifications, e.g., *N*‐methylation, or by developing fully non‐peptidic peptidomimetics, which have allowed producing ligands with high affinity and selectivity for closely related integrins, such as α_v_β_3_ and α_5_β_1_ [28, 29, 30, 166]. However, this strategy remains to be fully explored on BMP‐derived peptides. We have recently shown that the cyclization of DWIVA retains the activity of the original peptide [143], but a comprehensive analysis of how conformational modifications of BMP‐peptides impact their activity and selectivity toward BMPRs is largely missing.

From a translational perspective, although a significant effort has been made to find a safer, cheaper, and more efficient pharmaceutical replacement for BMP‐2, most advanced clinical systems still rely on the administration of supraphysiological concentrations of BMP‐2, and no suitable alternative has been identified that elicits similar or superior efficacy in inducing bone formation without the adverse effects associated with the use of BMP‐2. Peptide‐based systems could, in principle, simplify manufacturing, reduce dosing, and avoid the supraphysiological exposure associated with recombinant BMPs. For instance, the systems described in this review have shown osteogenic signaling at doses (generally in the pmol/cm^2^ range) much lower than the clinical standard, which consists of BMP‐2 loaded into collagen sponges at high concentrations (1.5 mg/mL). Another advantage is that the peptides are immobilized, thus avoiding systemic circulation and unwanted off‐target effects. However, none of the integrin‐GF‐mimetic peptide systems have yet progressed into regulatory pipelines. This is due to several reasons. (i) In the first place, additional evidence from animal studies is still needed to validate the potential of this strategy for clinical translation. Indeed, the evaluation of peptide‐based strategies in vivo is scarce, and the use of a BMP‐2 control to assess comparative efficacy is often missing. (ii) Second, the translation of peptide‐functionalized biomaterials will still require demonstration of scalable synthesis—under GMP‐compatible production–, long‐term stability and release testing, batch reproducibility, and regulatory compliance (drug‐device combination vs. product classification—class II/III implant).

Another relevant question concerns which integrin subtypes are involved in the synergistic crosstalk with GFs. In the specific case of BMP‐2, this synergy has been primarily attributed to α_5_β_1_ integrin—however, it is important to note that earlier studies commonly used FN (or its fragments), which co‐presents RGD alongside the PHSRN synergy site, thereby enhancing binding to this integrin subtype. Notably, studies employing linear RGD or the α_v_β_3_‐selective cyclic RGD peptide have also highlighted a significant role for α_v_β_3_ in mediating this synergy. In this regard, Fourel et al. reported that α_v_β_3_ integrin is required to mediate BMP‐2–induced Smad signaling through a Cdc42–Src–FAK–ILK pathway, demonstrating that BMPRs and β_3_ integrin cooperate to control focal adhesion dynamics and Smad signaling, thus directing cell migration and fate commitment [63]. Additionally, it is known that the co‐presentation of BMP‐2 and α_5_β1 integrin‐selective ligands facilitates the recruitment of α_v_β_3_ integrins in focal adhesions to stabilize their assembly and sustain forces. However, the underlying mechanisms of this process are still under investigation [96]. Likewise, the collagen‐derived GFOGER sequence, which specifically binds to α_2_β_1_, has demonstrated synergistic effects when combined with BMP‐2. This indicates that at least three different integrin subtypes are involved in synergistic signaling with BMP‐2. However, a common limitation is that the majority of studies in the field working with peptides rely on the use of linear RGD, which is not selective and binds to several integrins with different degrees of affinity. Even cyclic RGD, which has a major affinity for α_v_β_3_, is not fully selective and displays activity for α_5_β_1_ integrin in the nanomolar range [29]. Hence, future studies aiming at clearly dissecting the role of α_5_β_1_/α_v_β_3_ in BMP‐mediated signaling should focus on the use of highly selective ligands coupled to functional assays with blocking antibodies and/or co‐localization analysis [167]. Whether additional integrin subtypes relevant to bone biology are also capable of engaging in such crosstalk with BMP receptors remains to be fully elucidated.

In this review, we have focused on the role of cell‐adhesive ECM proteins and GFs in receptor crosstalk and osteogenesis. However, other critical cues must also be considered when regulating BMP signaling and osteodifferentiation. Among these, matrix stiffness and related mechanotransduction events represent particularly important biophysical factors [165, 168]. Integrin‐mediated mechanotransduction and its influence on cell adhesion and osteogenic differentiation are well‐characterized [60]; however, the impact of mechanical cues on BMP activity remains comparatively underexplored [169]. Emerging evidence suggests this area is highly relevant, as BMP‐2 signaling has recently been shown to be associated with mechanotransduction through the YAP/TAZ pathway [170]. Indeed, matrix mechanics strongly modulate the ability of cells to interpret BMP cues. Stiff substrates promote integrin‐dependent force transmission, focal adhesion maturation, and YAP/TAZ nuclear translocation, which in turn amplify SMAD1/5 signaling and osteogenic gene expression. Conversely, soft matrices reduce cytoskeletal tension, impair BMP receptor clustering, and attenuate SMAD phosphorylation even in the presence of exogenous BMP‐2. These findings highlight that BMP activity must be understood in the context of the mechanical microenvironment that governs receptor engagement and downstream transcriptional responses. In line with this, in a recent study, Picart and colleagues investigated cellular responses to four BMPs (BMP‐2, 4, 7, 9) on thin films with varying stiffness. Notably, cellular responses were both BMP‐specific and stiffness‐dependent, highlighting the complex interplay between biochemical and mechanical cues [171]. Future studies investigating the combined effects of biochemical signals (e.g., RGD, BMPs) and physical properties (e.g., substrate stiffness) on integrin‐GF signaling and differentiation will be essential for the rational design of next‐generation biomaterials for clinical repair of bone tissue.

Finally, there are other components of the ECM that also contribute significantly to the regulation of bone differentiation. For instance, cadherins are cell–cell adhesion molecules that form adherens junctions and participate in mechanosensing, intracellular signaling, and tissue organization. Valat et al. recently reported that BMP‐2 might play an important role in influencing cadherin and integrin‐dependent signals to control cell fate by regulating the strength of adhesion to the ECM, as well as ECM remodeling and mechanics [172], indicating that BMPs, integrins, and cadherins cooperate in osteogenic differentiation.

Glycosaminoglycans (GAGs), such as heparan sulfate and chondroitin sulfate, are another key ECM component that interacts with a wide range of signaling molecules, including BMPs, FGF, and VEGF. In particular, the binding affinity between GAGs and BMP‐2 depends on multiple factors, including the localization of the proteoglycans (either on the cell surface or within the ECM), the specific type of GAG, and the precise sulfation pattern. However, the exact mechanisms underlying these interactions remain elusive, and the role of GAGs in modulating BMP signaling is still relatively underexplored [173, 174, 175]. Advances in this area could have significant implications for the development of biomaterials that target BMP signaling pathways, influence stem cell fate decisions, and enhance osteoinductive outcomes. For instance, it was demonstrated that the co‐immobilization of heparan sulfate with cyclic RGD promoted BMP‐2‐mediated signaling and osteogenic differentiation, compared to BMP‐2 alone [176].

Incorporating these additional layers of ECM‐mediated signaling—beyond integrin–GF crosstalk—can offer a more comprehensive understanding of the cellular microenvironment and opens new avenues for the design of multifunctional biomaterials that more closely mimic the complexity of native bone tissue.

## Conflicts of Interest

The authors declare no conflicts of interest.

## Data Availability

The authors have nothing to report.
